# Self-adaptive hetero-phase superlattices in TaS_2_ via layer-resolved 1T-to-1H transformations

**DOI:** 10.1093/nsr/nwag246

**Published:** 2026-04-28

**Authors:** Zhenyu Ding, Yihao Wang, Rui Li, Jingjing Gao, Jialiang Jiang, Jin Tang, Yuyan Han, Qian Xu, Junfa Zhu, Wenqian Tu, Wenjian Lu, Yingguo Yang, Zhihao Li, Xingyu Gao, Zhe Qu, Yuping Sun, Xuan Luo, Xiaoping Yang, Hai Xu, Yimin Xiong, Liang Cao

**Affiliations:** Anhui Provincial Key Laboratory of Low-Energy Quantum Materials and Devices, High Magnetic Field Laboratory, HFIPS, Chinese Academy of Sciences, Hefei 230031, China; School of Optoelectronic Science and Engineering, Anhui University, Hefei 230601, China; Science Island Branch of Graduate School, University of Science and Technology of China, Hefei 230026, China; Anhui Provincial Key Laboratory of Low-Energy Quantum Materials and Devices, High Magnetic Field Laboratory, HFIPS, Chinese Academy of Sciences, Hefei 230031, China; Anhui Provincial Key Laboratory of Low-Energy Quantum Materials and Devices, High Magnetic Field Laboratory, HFIPS, Chinese Academy of Sciences, Hefei 230031, China; Science Island Branch of Graduate School, University of Science and Technology of China, Hefei 230026, China; Key Laboratory of Materials Physics, Institute of Solid State Physics, HFIPS, Chinese Academy of Sciences, Hefei 230031, China; Anhui Provincial Key Laboratory of Low-Energy Quantum Materials and Devices, High Magnetic Field Laboratory, HFIPS, Chinese Academy of Sciences, Hefei 230031, China; Department of Physics, School of Physics, Anhui University, Hefei 230601, China; Anhui Provincial Key Laboratory of Magnetic Functional Materials and Devices, Anhui University, Hefei 230601, China; Anhui Provincial Key Laboratory of Low-Energy Quantum Materials and Devices, High Magnetic Field Laboratory, HFIPS, Chinese Academy of Sciences, Hefei 230031, China; National Synchrotron Radiation Laboratory, University of Science and Technology of China, Hefei 230029, China; National Synchrotron Radiation Laboratory, University of Science and Technology of China, Hefei 230029, China; Key Laboratory of Materials Physics, Institute of Solid State Physics, HFIPS, Chinese Academy of Sciences, Hefei 230031, China; Key Laboratory of Materials Physics, Institute of Solid State Physics, HFIPS, Chinese Academy of Sciences, Hefei 230031, China; State Key Laboratory of Photovoltaic Science and Technology, School of Microelectronics, Fudan University, Shanghai 200433, China; Anhui Provincial Key Laboratory of Low-Energy Quantum Materials and Devices, High Magnetic Field Laboratory, HFIPS, Chinese Academy of Sciences, Hefei 230031, China; Yangtze Memory Technologies Co., Ltd., Wuhan 430205, China; Shanghai Synchrotron Radiation Facility (SSRF), Zhangjiang Laboratory, Shanghai Advanced Research Institute, Chinese Academy of Sciences, Shanghai 201204, China; Anhui Provincial Key Laboratory of Low-Energy Quantum Materials and Devices, High Magnetic Field Laboratory, HFIPS, Chinese Academy of Sciences, Hefei 230031, China; Anhui Provincial Key Laboratory of Low-Energy Quantum Materials and Devices, High Magnetic Field Laboratory, HFIPS, Chinese Academy of Sciences, Hefei 230031, China; Key Laboratory of Materials Physics, Institute of Solid State Physics, HFIPS, Chinese Academy of Sciences, Hefei 230031, China; Collaborative Innovation Center of Advanced Microstructures, Nanjing University, Nanjing 210093, China; Key Laboratory of Materials Physics, Institute of Solid State Physics, HFIPS, Chinese Academy of Sciences, Hefei 230031, China; Anhui Provincial Key Laboratory of Low-Energy Quantum Materials and Devices, High Magnetic Field Laboratory, HFIPS, Chinese Academy of Sciences, Hefei 230031, China; School of Optoelectronic Science and Engineering, Anhui University, Hefei 230601, China; State Key Laboratory of Opto-Electronic Information Acquisition and Protection Technology, Anhui University, Hefei 230601, China; Department of Physics, School of Physics, Anhui University, Hefei 230601, China; Anhui Provincial Key Laboratory of Magnetic Functional Materials and Devices, Anhui University, Hefei 230601, China; Hefei National Laboratory, Hefei 230028, China; Anhui Provincial Key Laboratory of Low-Energy Quantum Materials and Devices, High Magnetic Field Laboratory, HFIPS, Chinese Academy of Sciences, Hefei 230031, China

**Keywords:** artificial superlattices, superconductivity, inter-phase coupling, stacking sequence, sliding

## Abstract

Artificial hetero-phase superlattices constructed from transition metal dichalcogenides (TMDs) provide a powerful platform for exploring exotic physical phenomena and delivering structurally robust devices. However, achieving deterministic control over phase-stacking sequences in bulk architectures remains a significant challenge. Here, we report a self-adaptive superlattice system formed in TaS_2_ crystals through an *in-situ* structural phase transition. Coordinated inter-layer sliding and intra-layer S-plane sliding drive layer-resolved 1T-to-1H transformations. This two-dimensional transformation pathway enables deterministic and dynamic engineering of hetero-phase sequences within a three-dimensional (3D) crystal, with the resulting interfaces stabilized by persistent inter-phase coupling. Within these reconfigurable superlattices, we identify two distinct superconducting states arising from paired 1H/1T bilayers and sandwiched 1H/1T/1H’ trilayers. The charge density wave order remaining in the 1T layer suppresses superconductivity in the 1H/1T superlattice. Our findings establish an *in*-*situ*, sequence-controllable phase engineering strategy for constructing bulk TMD hetero-phase homostructures and highlight stacking configuration as a powerful degree of freedom for designing TMD-based quantum materials and devices.

## Teaser

Van der Waals materials can reconfigure adaptively into programmable superlattices, enabling tunable superconductivity solely through structural control—a new route to on-demand quantum material engineering.

## INTRODUCTION

Unlike graphene, monolayer transition metal dichalcogenides (TMDs) possess an intrinsic triple-atomic-plane structure in which a transition-metal plane is sandwiched between two chalcogen planes (MX_2_). This structural motif accommodates diverse coordination geometries and permits multiple intrinsic polymorphs, each associated with distinct electronic states, including charge density waves (CDWs), Mott-insulating states, superconductivity, and topological states [1–3]. TMD materials typically exhibit two fundamental monolayer polymorphs. For instance, the octahedral 1T-TaS_2_ phase, characterized by CDW-induced David-star distortions, has been proposed as a Mott-insulator with narrow flat bands near the Fermi level (*E*_F_, Fig. S1a and b) [4], potentially hosting a quantum spin liquid ground state [5]. In contrast, the trigonal prismatic 1H-TaS_2_ phase is metallic (Fig. S1c) and displays unconventional superconductivity [6,7].

Motivated by the success of van der Waals (vdW) ‘LEGO-type’ heterostructures assembled from two-dimensional (2D) materials [8], artificially stacking distinct TMD polymorphs has emerged as a powerful approach for tuning electron correlations, charge ordering, and topological behavior [9–12]. At the bulk level, naturally

occurring 4H- and 6R-polytypes exhibit alternating 1H and 1T stacking sequences [13–22], and demonstrate how hetero-phase stacking, despite identical chemical composition, can stabilize distinct superconducting or correlated states. For example, 4H_b_-TaS_2_ crystals exhibit chiral superconductivity with a superconducting transition onset temperature (*T*_C_) of 2.7–3.8 K [21–28], whereas 6R-TaS_2_ crystals display nematic superconductivity with *T*_C_ ranging from 1.9 to 3.3 K [15–17]. These distinct phenomena underscore the central role of stacking geometry and inter-phase coupling in governing emergent electronic states.

Artificially engineered hetero-phase superlattices in bulk TMDs would thus offer a deterministic route to probe stacking-dependent quantum states, while simultaneously expanding the design space for robust, multifunctional architectures [29]. However, unlike vdW heterostructures assembled *ex*-*situ*, realizing phase-defined superlattices within a three-dimensional (3D) crystal requires atomic-scale precision, defect-free interfaces, and preservation of structural integrity over macroscopic distances [8]. Conventional exfoliation-restacking or layer-by-layer chemical vapor deposition remains inadequate for achieving such control [1,30]. In contrast, *in*-*situ* phase transitions in TMDs crystals offer a promising route to realize hetero-phase homostructures without introducing impurities or defects [31–35]. Although substantial progress has been made in monolayer or few-layer systems [35–38], it remains unclear whether layer-resolved transformations can be sustained deep within bulk TMDs crystals [31,32], where increased electrostatic and elastic interactions reshape the local energy landscape.

Here, we address this challenge by demonstrating an *in-situ*, self-adaptive phase-engineering strategy in bulk 1T-TaS_2_. We show that controlled inter-layer sliding weakens inter-layer coupling and enables intra-layer S-plane sliding, triggering deterministic 1T-to-1H transitions on a layer-by-layer basis. The resulting inter-phase coupling stabilizes multiple stacking configurations, allowing the formation of three classes of self-adaptive superlattices. Systematic transport measurements reveal two prototypical superconducting units of paired 1H/1T bilayers and sandwiched 1H/1T/1H′ trilayers (1H′ denotes the 60° rotational variant of the 1H phase), each hosting distinct superconducting states. These results establish stacking sequence, independent of chemical composition, as a fundamental degree of freedom for tuning quantum phenomena in TMDs. Our study introduces a generalizable *in-situ* phase engineering framework for constructing stable, reconfigurable hetero-phase superlattices with tunable electronic functionalities.

## RESULTS

### *In-situ* structural phase transitions and the formation of self-adaptive superlattices

Structural transformation in bulk crystals, including structural phase transitions and the structural reconstruction processes coupled with hetero-phase stacking-order rearrangements constitute a dynamic, continuous, and complex evolution process. To capture this evolution, we adopted a snapshot strategy to assemble a panoramic view of the transformation pathway. By freezing the stepwise structural development, a series of 1T-TaS₂ crystals were annealed separately at different temperatures. The atomic-scale phase transition and emergent hetero-phase stacking configurations were systematically examined across five distinct annealing temperature windows using aberration-corrected high-angle annular dark-field scanning transmission electron microscopy (HAADF-STEM).

**Figure 1.**
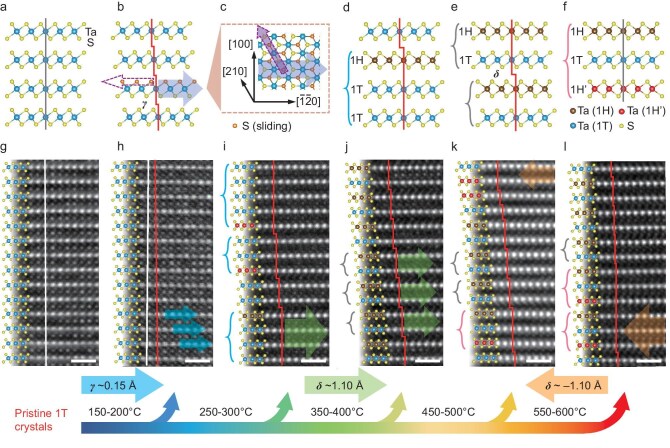
Thermally driven *in*-*situ* phase transition in bulk crystals and hetero-phase superlattices. (a) Schematic side view of 1T-TaS_2_. (b) Collective inter-layer sliding in LC-TaS_2_ and illustration of opposing intra-layer S-plane sliding, which triggers 1H transitions. (c) Basic plane view of a seed layer undergoing the 1T-to-1H transition, facilitated by top S-plane sliding along [210] (narrow dashed arrow) and inter-layer sliding along $[ {\bar{1}\bar{2}0} ]$ (broad transparent arrow). (d–f) Schematic representations of superlattice units of (d) 1H/multilayer-1T, (e) paired 1H/1T and (f) sandwiched 1H/1T/1H′ with vertical Ta-Ta alignment guided by braces. [100] cross-sectional HAADF-STEM images of (g) pristine 1T-TaS_2_, (h) LC-TaS_2_, (i) 1H/multi-1T(i)-TaS_2_, (j) 1H/1T-TaS_2_, (k) 1H/1T/1H′(i)-TaS_2_, and (l) 1H/1T/1H′(iii)-TaS_2_ (Scale bar, 1 nm). White vertical lines in panels (g, h) indicate the out-of-plane direction. Inclined line and polylines in panels (h–l) highlight the inter-layer ***γ*-** and inter-phase ***δ*-**misalignments, respectively. The arrows guide static sliding along $[ {\bar{1}\bar{2}0} ]$ or [120]. Bright spots in the vdW gaps are attributed to Ga-atoms introduced during focused ion beam processing for STEM sample preparation, rather than self-intercalated Ta-atoms (note S3).

At moderate annealing temperatures of 150–200°C, LC-TaS_2_ crystals exhibit collective inter-layer sliding with a 40-layer periodicity (Fig. 1h and Fig. S2) and a translational misalignment of ***γ*** = ***a***√3/40 (where ***a*** is the in-plane lattice vector), consistent with previous reports [2]. This collective motion precedes the onset of individual-layer 1T-to-(1H or 1H′) transitions, which emerge after 250–300°C annealing (Fig. 1i and Fig. S3). The newly formed 1H or 1H′ layer acts as a seed layer that restores the vertical Ta-Ta alignment with the remaining 1T multilayer (multilayer-1T), reminiscent of pristine 1T-TaS_2_ crystals (Fig. 1a and g). This reconstruction simultaneously generates inter-phase misalignment above (below) the 1H (1H′) seed layer (misaligned 1T-1H or 1H′-1T). Crystals hosting the 1H/multilayer-1T superlattice (Fig. 1d), in which each unit is defined by vertical Ta-Ta alignment, are named as 1H/multi-1T(i)-TaS_2_, where ‘(i)’ denotes the initial transformation stage.

Direct annealing at intermediate 350–400°C produces paired 1H/1T hetero-phase bilayers and a periodically laddered stacking configuration along the out-of-plane direction (Fig. 1j). Large-area STEM image (Fig. S4) reveals a 16-layer periodicity, corresponding to a translational misalignment of ***δ*** = ***a***3√3/16. This artificial 1H/1T superlattice (Fig. 1e) differs from previously reported natural 6R-TaS_2_, which exhibits a 6-layer periodicity [15–18], and is denoted as 1H/1T-TaS_2_. Recent reports suggest that self-intercalated Ta-atoms in vdW gaps stabilize the characteristic 6-layer periodicity of 6R-TaS_2_ [39], which may explain the distinct stacking periodicity in the absence of intercalated species (note S3). A similar stabilization mechanism *via* additional atomic substitution has also been reported in 6R-NbSe_2_ [19]. Such variations in stacking periodicity are expected to modify inter-layer coupling and electronic properties, as subtle translational shifts in 1T-TaS_2_ can drive a transition from a 3D band insulator to a 2D Mott insulator [2].

At higher annealing temperatures (450–500°C), sandwiched 1H/1T/1H′ units emerge (Fig. 1k and f), forming crystals designated as 1H/1T/1H′(i)-TaS_2_. Promoting the temperature to 550–600°C, the sandwiched units become more abundant (Fig. 1l and Fig. S5), accompanied by the elimination of ***δ***-misalignments, yielding 1H/1T/1H′(iii)-TaS_2_ crystals. The ‘(iii)’ designation marks a more advanced transformation stage.

The spatial uniformity of both paired 1H/1T and sandwiched 1H/1T/1H′ configurations was verified by STEM images acquired from regions separated by millimeter-scale distances within the same 1H/1T-TaS_2_ and 1H/1T/1H′(iii)-TaS_2_ crystals. Consistent structural features observed demonstrate a high degree of structural homogeneity across macroscopic length scales (Figs S4 and S5). Additional Raman spectra were also collected to verify the structural transformation, as shown in Fig. S6.

### Structural transformation pathway and DFT calculated energetics

**Figure 2.**
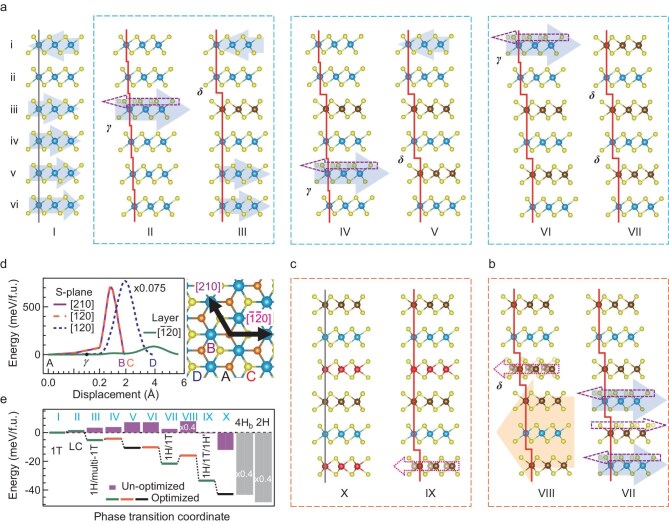
Structural transformation pathway and corresponding DFT energy landscapes. (a) Schematic illustration of the transformation from pristine 1T-TaS_2_ (I) through inter-layer sliding (II) and to intermediate 1H/multi-1T (III) and paired 1H/1T (VII) superlattices, accompanied by the generation of inter-phase ***δ*-**misalignment. This process is driven by coordinated inter-layer sliding and intra-layer S-plane sliding, with repeatability highlighted by dashed boxes. (b and c) Further transformation into sandwiched 1H/1T/1H′ superlattices occurs through additional 1T-to-1H transitions, stacking order reconstruction, and 1H-to-1H′ transitions, ultimately restoring vertical Ta-Ta alignment and eliminating ***δ*-**misalignment. The layer (ii) in panels (a–c) remains stationary. Broad transparent arrows indicate the inter-layer sliding, while narrow dashed arrows mark intra-layer atomic-plane sliding that triggers structural phase transitions. (d) DFT-calculated energy profiles for inter-layer sliding along $[ {\bar{1}\bar{2}0} ]$ in a bilayer system, and for intra-layer S-plane sliding along [210], $[ {\bar{1}\bar{2}0} ]$, and [120] directions in a ***γ*-**misaligned bilayer. (e) Corresponding energy profile for various stacking sequences (I-X) before and after out-of-plane structural optimization, with well-defined 4H_b_- and 2H-phase stacking included for comparison.

STEM observations reveal a well-defined sequence of structural transformations in TaS_2_ crystals. (i) Collective inter-layer sliding occurs first, preceding local structural phase transitions. (ii) Individual-layer 1T-to-(1H or 1H′) transitions follow, suppressing inter-layer sliding of residual 1T layers on one side while developing inter-phase stacking misalignment on the other. The newly formed 1H or 1H′ layer maintains registry with adjacent 1T layer(s), yielding intermediate 1H/multilayer-1T, and paired 1H/1T superlattices. (iii) Further 1H′ transitions and structural reconstruction eliminate inter-phase misalignments, resulting in sandwiched 1H/1T/1H′ superlattices. To model these processes, a series of representative stacking configurations in a 6-layer slab system was proposed (Fig. 2a–c), tracing the evolution from pristine 1T-TaS_2_ (I) to intermediate 1H/multilayer-1T (III), paired 1H/1T superlattices (VII), and finally sandwiched 1H/1T/1H′ superlattices (X). Density functional theory (DFT) calculations were performed both before and after out-of-plane structural optimization to evaluate the total energies of each configuration (Fig. 2e). This approach allows the structural evolution to be conceptually decomposed into two steps: (i) the phase transformation and (ii) the subsequent inter-layer relaxation as revealed by synchrotron-based X-ray diffraction (Fig. S10), highlighting the critical role of inter-layer relaxation in determining stacking stability. The calculated energy difference between 1T-TaS_2_ and thermodynamically stable 2H-TaS_2_ references is 121.27 meV/f.u., consistent with literatures [38,40].

The energy evolution as a function of sliding displacement in a bilayer system was also calculated (Fig. 2d). The energy barriers for intra-layer S-plane sliding along long diagonal [210] and $[\bar{1}\bar{2}0 ]$ directions are ∼700 meV/f.u., corresponding to S-atoms occupying bridge positions, consistent in magnitude with reported value for monolayer MoS_2_ [41]. Inter-layer sliding along $[ {\bar{1}\bar{2}0} ]$ requires a significantly lower barrier than intra-layer S-plane sliding. This reflects the intrinsic combination of inter-layer slipperiness and intra-layer stiffness characteristic of TMDs crystals, and explains the experimentally observed preference for collective inter-layer sliding (Fig. 1h and configuration II in Fig. 2a) over local 1T-to-(1H or 1H′) transitions (Fig. 1i and configuration III in Fig. 2a).

As illustrated in Fig. 2a, initial inter-layer ***γ***-sliding (I→II) slightly raises the system energy to 1.02 meV/f.u. after out-of-plane direction optimization (Fig. 2e). Subsequent intra-layer S-plane sliding drives the individual-layer 1T-to-1H transition (II→III). The transformed 1H-TaS_2_ seed layer (iii in III), suppresses collective inter-layer motion and introduces inter-phase ***δ***-misalignment, which lowers the energy to –5.25 meV/f.u. after optimization (Fig. 2e). The kinetic barrier associated with configuration (II) stabilizes the intermediate 1H/multilayer-1T superlattice against back-transformation. Notably, under inter-layer ***γ***-sliding along $[ {\bar{1}\bar{2}0} ]$, intra-layer S-plane sliding preferentially occurs along energetically favorable [210] or symmetry-equivalent $[ {\bar{1}10} ]$ directions (705.49 meV/f.u.) rather than along [120] or $[ {\bar{1}\bar{2}0} ]$ (717.35 meV/f.u., Fig. 2d and Fig. S11) [36,42]. This opposing directional preference between inter-layer and intra-layer sliding plays an essential role in determining the phase transition mechanism in bulk, as discussed later. Repeated 1T-to-1H transitions (IV→V and VI→VII), coordinated with inter-layer sliding (IV and VI) and intra-layer S-plane sliding (V and VII), yield paired 1H/1T units with periodic ***δ***-misalignment (VII). The system energy rises to 7.19 meV/f.u. for intermediate configurations (V and VI), then decreases to 2.58 meV/f.u. for configuration (VII) prior to structural optimization, and further drops to –21.64 meV/f.u. after optimization, highlighting the structural stability of the paired 1H/1T superlattice.

As illustrated in represented configuration (VII) in Fig. 2b, further inter-layer sliding of 1T layers (iv and vi) decouples the paired 1H/1T units. Subsequent reconstruction reverses the stacking from misaligned 1T-1H (iv and v) to a paired 1H/1T unit (VII→VIII), increasing system energy regardless of structural optimization (Fig. 2e). This kinetic barrier further stabilizes the paired 1H/1T superlattice. The reconstructed configuration (VIII) generates a split 1H layer (iii). This layer undergoes a 1H-to-1H′ transition (VIII→IX) *via* intra-layer Ta-plane sliding, creating a vertically aligned 1H/1T/1H′ unit. Owing to the reduced repulsion between newly formed 1H′ layer (iii in IX) and the underlying 1H layer (iv in IX), the paired 1H/1T units can translate collectively (broad transparent arrow in VIII), eliminating the ***δ***-misalignment and reducing the system energy. This process repeats, producing another split 1H layer (vi in IX), which also transitions to 1H′ phase (IX→X), ultimately forming a sandwiched 1H/1T/1H′ superlattice free of stacking misalignments. The optimized structure exhibits a relatively low energy of –42.85 meV/f.u. (Fig. 2e), corresponding to a metastable local minimum. However, the likely absence of a substantial energy barrier toward the energetically more favorable 4H_b_ configuration implies that this phase is only metastable and can readily relax to the global minimum, making the realization of a pure 1H/1T/1H′ superlattice (X) challenging.

Phonon dispersion calculations further confirm the stability of both vertical Ta-Ta aligned 1H/1T bilayer and 1H/1T/1H′ trilayer configurations under annealing-relevant conditions of 350–600°C (Fig. S12). Using electronic smearing values (*σ* = 0.01, 0.2, and 0.4 eV) to approximate finite-temperature effects, imaginary modes present at *σ* = 0.01 eV and disappear at higher *σ*, consistent with previous studies of thermally stabilized 6R-TaS_2_ [15]. The low-temperature instability originates from CDW-distortions, which are suppressed at elevated temperatures, confirming the dynamic stability of hetero-phase superlattices during thermal processing.

### Inter-phase coupling in hetero-phase superlattices

**Figure 3.**
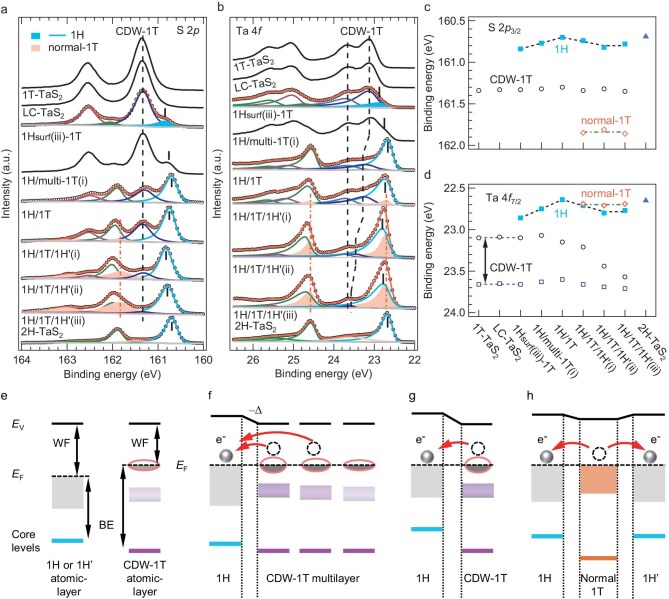
Structural phase transition and inter-phase coupling in hetero-phase superlattices. (a) Synchrotron-based S 2*p* and (b) Ta 4*f* core-level spectra and corresponding fitting results for pristine 1T-TaS_2_ and phase-engineered TaS_2_ crystals, along with 2H-TaS_2_ reference. The evolution of (c) S 2*p*_3/2_ and (d) Ta 4*f*_7/2_ binding energies extracted from panels (a, b), highlighting binding energy shifts and reduced CDW-splitting associated with hetero-phase formation. The dashed (doted) lines are guided to the eye. (e) Schematic band structures of 1H or 1H′ atomic-layer and CDW-1T atomic-layer. Band alignments illustrating inter-phase charge transfer at (f) 1H/CDW-1T multilayer, (g) 1H/CDW-1T, and (h) 1H/normal-1T/1H′ interfaces, corresponding to 1H/multilayer-1T, 1H/1T, and 1H/1T/1H hetero-phase superlattice units, respectively.

To understand the role of inter-phase charge transfer in governing the formation and stabilization of distinct hetero-phase superlattices, synchrotron-based photoemission spectroscopy (PES) was performed at room temperature. Except for 1H_surf_(iii)-1T-TaS_2_, which hosts a surface-limited phase transition (Fig. S10 and note S4) [38], all crystals were cleaved to expose bulk-representative surfaces. In 1T-TaS_2_ crystals, where 1T layers host the nearly-commensurate CDW state (CDW-1T), the S 2*p* doublet exhibits a dominant S 2*p*_3/2_ peak at ∼161.34 eV (Fig. 3a). The David-star distortion characteristic of CDW states (Fig. S1b) induces charge redistribution within each David-star cluster, resulting in unequal local electron density at specific Ta-sites and thus a further CDW-induced splitting of the Ta 4*f* doublet. Two well-resolved Ta 4*f*_7/2_ components appear at ∼23.64 and ∼23.10 eV (Fig. 3b, dashed lines), consistent with previous reports [2,43]. Inter-layer sliding in LC-TaS_2_ crystals has negligible influence on these binding energies (BEs). The 1H layer in 1H_surf_(iii)-1T-TaS_2_, 1H/multi-1T(i)-TaS_2_ and 1H/1T-TaS_2_ crystals gives rise to additional lower BE features in both S 2*p* and Ta 4*f* spectra (Fig. 3a and b, solid bars). The lower BE characteristics, reminiscent of 2H-TaS_2_ crystals, are attributed to enhanced core-hole screening due to the increased local electron density in 1H or 1H′ layers [43]. Upon formation of sandwiched 1H/1T/1H′ units in three 1H/1T/1H′(i/ii/iii)-TaS_2_ crystals, new S 2*p* and Ta 4*f* features emerge, marked by opposite BE shift. Specifically, the S 2*p* shift to a higher BE (∼161.8 eV for S 2*p*_3/2_), while the Ta 4*f* shifts to a lower BE (∼22.7 eV for Ta 4*f*_7/2_), as guided by dashed-dot lines in Fig. 3a and b. Simultaneously, the CDW-1T component weakens, implying a transition from the CDW-distorted-1T to undistorted normal-1T layer. The increased intensity of corresponding normal-1T signal reflects the growing fraction of 1H/1T/1H′ units from 1H/1T/1H′(i)-TaS_2_ to 1H/1T/1H′(iii)-TaS_2_ crystals.

The evolution of core-levels BEs (Fig. 3c and d) provides insight into inter-phase coupling. Surface 1H layer in 1H_surf_(iii)-1T-TaS_2_ exhibits S 2*p*_3/2_ at 160.85 eV and Ta 4*f*_7/2_ at 22.85 eV, higher than those in 2H-TaS_2_ crystals. This implies net electron donation. Both levels shift to lower BEs for internal 1H or 1H′ layers in 1H/multi-1T(i)-TaS_2_, and reach a minimum of 160.70 and 22.65 eV in 1H/1T-TaS_2_, indicative of reduced electron donation. In the CDW-1T layer(s) counterpart, CDW-induced Ta 4*f* splitting decreases from ∼0.54 eV in 1H_surf_(iii)-1T-TaS_2_ to ∼0.45 eV in 1H/1T-TaS_2_ (Fig. 3d), indicating CDW amplitude softening due to reduced electron density within the CDW-clusters. These observations point to electron transfer from CDW-clusters in CDW-1T layer(s) to the paired 1H layer, consistent with the theoretical calculation [9,44].

The inter-phase charge redistribution is governed by interfacial band alignment, as illustrated in Fig. 3e–g. In a CDW-1T atomic-layer, each David-star cluster comprises 13 Ta-atoms, each contributing one 5*d* electron. Twelve paired electrons contribute to lower band, whereas one unpaired electron responses to a narrow half-filled band at *E*_F_ (Fig. 3e) [45]. At the 1H/CDW-1T multilayer interface (Fig. 3f), which are present in 1H_surf_(iii)-1T-TaS_2_ and 1H/multi-1T(i)-TaS_2_ crystals, Fermi level misalignment induces electron transfer from CDW-1T multilayer to the 1H atomic-layer until equilibrium is established. A built-in dipole (Δ) is expected to prevent further electron transfer. As the CDW-1T multilayer thin down to single-layer dimensions (Fig. 3g), less electrons transfer results in a reduced Fermi level shift in the 1H atomic-layer. Accordingly, the lowest core-levels BEs for 1H layers are reached in 1H/1T-TaS_2_ crystals (Fig. 3c and d). Meanwhile, the almost constant S 2*p* BE of residual CDW-1T layer(s) (Fig. 3c) indicates an unshifted Fermi level in CDW-1T layer(s), as illustrated in Fig. 3e–g. However, the electron depletion softens the CDW amplitude, as evidenced by the reduced CDW-splitting of the Ta 4*f* peaks. This softening likely broadens the narrow band at *E*_F_ (Fig. 3f and g), due to reduced on-site Coulomb interactions [46].

In sandwiched 1H/1T/1H′ superlattice of all three 1H/1T/1H′(i/ii/iii)-TaS_2_ crystals, the higher BE shift of 1H and 1H′ layers core-levels (Fig. 3c and d) implies additional electrons transfer to 1H and 1H′ layers (Fig. 3h) upon Ta-Ta alignment. This suggests that the inter-phase misalignment in paired 1H/1T superlattice hinders electron transfer from 1T to misaligned 1H layers due to reduced orbital overlap. Enhanced electron depletion in the intervening 1T layer drives the collapse of CDW states, as evidenced by emergence of normal-1T components and significantly reduced CDW-splitting of Ta 4*f* (Fig. 3b and d). Such CDW collapse has been reported in 4H_b_-TaS_2_ crystals, where the 1T layer is sandwiched between 1H- and 1H′ layers [47]. Saturation occurs once 1H/1T/1H′ units dominate in 1H/1T/1H′(iii)-TaS_2_.

Together, these results reveal that the inter-phase charge transfer is a key mechanism stabilizing 1H/multilayer-1T, 1H/1T and 1H/1T/1H′ hetero-phase superlattices.

### Insulating-to-superconducting electronic phase transition

**Figure 4.**
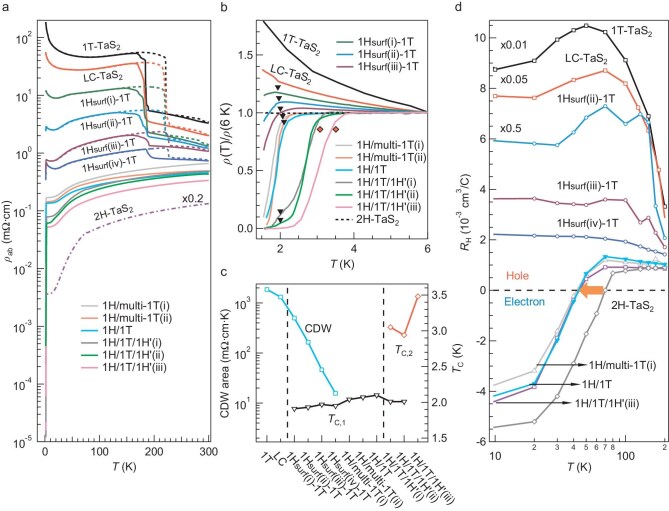
Electrical transport properties of phase-engineered TaS_2_ crystals. (a) Temperature-dependent in-plane resistivity *ρ*_ab_ and (b) close-up of resistivity near the superconducting transition. In panel (b), all curves are normalized to the resistivity at 6 K for clarity. (c) The extracted superconducting transitions temperatures (*T*_C,1_, *T*_C,2_), and relative hysteresis area associated with CDW transitions (CDW area). (d) The temperature dependence of Hall coefficient *R*_H_ for representative phase-engineered TaS_2_ crystals, highlighting carrier-type evolution and inter-phase charge transfer effects.

To explore the macroscopic consequences of phase engineering in TaS_2_ crystals, the electrical transport measurements were conducted. Figure 4a represents the temperature dependence of in-plane resistivity (*ρ*_ab_) for a series of phase-engineered crystals, including the as-grown 2H-TaS_2_ reference. The pristine 1T-TaS_2_ crystal displays overall insulating behavior with a pronounced hysteresis loop associated with commensurate and near commensurate CDW transitions. The LC-TaS_2_ exhibits lower resistivity values and a slightly reduced hysteresis area. In four 1H_surf_(i/ii/iii/iv)-1T-TaS_2_ crystals, both resistivity and hysteresis area decrease systematically, whereas the CDW transition temperatures remain unchanged. At lower temperature, a sharp drop in resistivity (Fig. 4a and b), which is suppressed by applied magnetic fields (Fig. S15), signals the emergence of superconductivity from the surface 1H layer. The gradual reduction of hysteresis area reflects the increasing surface superconducting volume fraction with lower overall resistivity (Fig. 4c). In these crystals, CDW order, insulating behavior, and surface superconductivity coexist. For bulk hetero-phase superlattice in 1H/multi-1T(i/ii)-TaS_2_ and 1H/1T-TaS_2_ crystals, the hysteresis disappears and the resistivity presents metallic behavior in the whole temperature range (Fig. 4a). A superconducting onset (*T*_C,1_) appears at ∼2.0 K (Fig. 4b and c), consistent with that of few-layer 6R-TaS_2_ (∼1.94 K) [16]. Sandwiched 1H/1T/1H′ superlattice units introduce a secondary *T*_C,2_ at 2.9–3.5 K, comparable to 2.7–3.8 K in 4H_b_-TaS_2_ crystals [21–27], and ∼3.4 K in monolayer 1H-TaS_2_ [6,48]. Both transitions are suppressed by magnetic fields (Fig. S15), confirming their superconducting origin.

Zero-field-cooled magnetic susceptibility under an in-plane magnetic field to minimize the demagnetization effect yields superconducting volume fractions of 58.5% for 1H/1T-TaS_2_ and 10.4% for 1H/1T/1H′(iii)-TaS_2_ (Fig. S16). Current density–voltage (*J*-*V*) characteristics collected at 1.6 K reveal a higher critical current density in 1H/1T/1H′(iii)-TaS_2_ than in 1H/1T-TaS_2_ (Fig. S17). Superconducting transitions were reproducible and ambient stability across five independent crystals, consistently showing *T*_C,1_ of 2.2–2.3 K for 1H/1T-TaS_2_, and *T*_C,2_ of 3.4–3.5 K for 1H/1T/1H′(iii)-TaS_2_ (Fig. S18). These results highlight the crucial role of stacking sequences in tuning superconductivity.

To assess the evolution of density of states and its interplay with structural transformations, the Hall coefficient *R*_H_ was extracted from linear fitting of Hall resistivity (*ρ*_xy_, Fig. S19). Figure 4d shows the temperature dependence of *R*_H_. In 2H-TaS_2_ reference, *R*_H_ switches from positive to negative at 3 × 3 CDW transition of ∼70 K, indicating electron-carriers dominated electrical transport at low temperature [48]. In contrast, *R*_H_ in 1T-TaS_2_ crystals remains positive below 200 K. These observations align well with the literatures [49,50]. In 1H_surf_(ii/iii/iv)-1T-TaS_2_ crystals, *R*_H_ decreases monotonically with increasing surface 1H layer fraction, indicating the general increase of the hole concentration due to electron depletion from dominant electrical transport 1T layers. For 1H/multi-1T(i)-TaS_2_, 1H/1T-TaS_2_ and 1H/1T/1H′(iii)-TaS_2_ crystals, *R*_H_ shows a clear sign reversal from positive to negative between 40 and 50 K, similar to 2H-TaS_2_ (∼70 K) but at a lower temperature. This implies that the 1H or 1H′ layers, which contribute electrons to the system, dominate electrical transport. Although the magnitude of *R*_H_ is comparable among three samples, making quantitative charge carrier density analysis challenging, the overall suppression of CDW transition relative to 2H-TaS_2_ reflects increased electron densities due to electron transfer doping of 1H or 1H′ layers. The tunability of CDW transition temperature, which can be reduced to ∼22 K in 6R- and 4H_b_-TaS_2_ crystals [16,24], is closely linked to the ability to control charge carrier density. This finding highlights the significant capacity for precise manipulation of charge carrier density in its 2D counterpart, such as through electronic doping or interface engineering, to fine-tune electronic states.

The enhancement of the superconducting transition temperature (∼2.1 K for 1H/1T-TaS_2_ crystals and ∼3.5 K for 1H/1T/1H′(iii)-TaS_2_ crystals) compared with 2H-TaS_2_ can be primarily attributed to the inter-layer decoupling of superconducting 1H and/or 1H′ layers by an intervening 1T layer. Potential contribution from defects, such as Ta-intercalation or S-vacancies [51], which would result in additional lower BE Ta-related features in the Ta 4*f* spectra (Fig. 3b) and deviations from the Ta:S stoichiometry (Fig. S8), can be ruled out. The concurrent suppression of the CDW order and enhancement of superconductivity supports the competing nature of these two electronic orders, closely resembling the behaviors reported in naturally occurring 6R- and 4H_b_-TaS_2_ crystals [16,24].

## DISCUSSION

### Layer-resolved 1T-to-1H transformations

The 2D 1T-to-1H transformation occurring within 3D 1T-TaS_2_ crystals can be rationalized by a two-tier sliding mechanism, involving coordinated inter-layer and intra-layer atomic-plane motion. This cooperative process differs fundamentally from the sole atomic-plane sliding typical of monolayer TMDs [36]. In bulk TaS_2_, collective inter-layer sliding increases long-range elastic energy and weakens inter-layer coupling, thus preventing global structural transformations. Instead, short-range electrostatic attraction between the transforming layer and the underlying layer facilitates the ongoing translational shift. The motion ceases when Pauli repulsion between the top S-atoms of the transforming layer and bottom S-atoms of the overlying layer becomes dominant, leading to strain accumulation at the sliding interface (Fig. 1b). This strain is released through top S-plane sliding in an opposing direction relative to the inter-layer sliding (Fig. 1c), triggering the 1T-to-1H transition at the atomic-layer level (Fig. 1d). The newly formed 1H seed layer subsequently drives successive transformations by restoring vertical Ta-Ta alignment with underlying 1T multilayer *via* inter-phase charge transfer. The resulting inter-phase misalignment with overlying 1T layer mitigates Pauli repulsion by avoiding unfavorable on-top S-S configurations. A symmetry-related pathway involving bottom S-plane sliding yields the 1H′ phase (Fig. S20).

This two-tier sliding underpins 2D structural phase transition in 3D crystals, enabling the formation of self-adaptive superlattices. Repetitive individual-layer 1T-to-1H transitions generate 1H/multilayer-1T architectures, and further evolve into paired 1H/1T (Fig. 1e) stabilized by electron transfer from 1T to 1H layers. Sandwiched 1H/1T/1H′ (Fig. 1f) can emerge either from simultaneous 1T-to-1H and 1T-to-1H′ transitions following collective inter-layer sliding in pristine 1T-TaS_2_ crystals (Fig. S20), or from stacking reconstruction of preformed paired 1H/1T superlattices (Fig. 2b and c). Electron transfer from the intervening 1T layer to the adjacent 1H and 1H′ layers further stabilizes the trilayer configuration.

The proposed two-tier sliding framework provides a unified description of *in-situ* structural phase transitions in bulk TMDs, capturing how the interplay of inter-layer and inter-phase couplings deterministically drives formation of hetero-phase superlattices. Extending this phase engineering strategy to other TMDs depends critically on inter-layer coupling strength. TMDs with moderate inter-layer coupling are ideal for achieving metastable hetero-phase architectures governed by inter-phase coupling. In contrast, TMDs with intrinsically weak inter-layer coupling, such as 2H-NbSe_2_ or 1T-TiS_2_ which naturally favor a single structural phase, are unlikely to undergo such transformations.

It is worth noting that the 1H/1T superlattice is non-centrosymmetric and hosts an interfacial dipole. In addition, the mirror configuration can be realized through the Ta-plane sliding induced 1H-to-1H′ transition, as illustrated in Fig. S21 and note S8. The satisfying on fundamental criterion for sliding ferroelectrics makes it a potential candidate for sliding ferroelectricity, deserving further investigation.

### Stacking-sequence-dependent superconductivity

Compared with conventional bottom-up techniques, *in-situ* phase engineering in bulk crystals offers notable advantages, including scalability, structural stability, atomically sharp interface, and deterministic stacking control over macroscopic (millimeter-scale) dimensions. Importantly, the precise control of stacking sequences introduces stacking order as an additional degree of freedom for tuning superconducting states, primarily through the modulation of inter-phase coupling. Among three superlattice architectures, the 1H/1T/1H′ configuration exhibits the highest superconducting transition temperature (∼3.5 K), comparable to monolayer 1H-TaS_2_. This configuration preserves global inversion symmetry, resembling the natural 4H_b_-polytype. The spatial separation between 1H and 1H′ layers, together with the collapse of CDW state in the intervening 1T layer due to enhanced electron depletion associated with electron transfer from 1T layer to the adjacent 1H and 1H′ layers, promotes intra-layer superconducting coherence. Furthermore, Ising spin–orbit coupling stemming from local inversion symmetry breaking in the 1H or 1H′ layer provides a favorable environment for exotic superconducting states [6,48], such as Fulde–Ferrell–Larkin–Ovchinnikov states [52], which were recently reported in 4H_b_-TaS_2_ [25].

In contrast, the 1H/1T superlattice, lacking global inversion symmetry and resembling the 6R-polytype, exhibits a lower transition temperature (∼2.1 K). Here, the 1T layer retains CDW order and transitions into Mott-insulating states at low temperatures, generating localized spins. Interactions between localized spins in the 1T layer and itinerant electrons in the 1H layer can suppress superconductivity, as demonstrated in artificial 2D 1H/1T-TaS_2_ homo-bilayer [10], and may induce hidden spin polarization, potentially giving rise to emergent phenomena such as chiral superconductivity, or nematic superconductivity as observed in 6R-TaS_2_ flakes [16]. It is therefore reasonable to conclude that the localized spins associated with the persistent CDW order in the 1T layer of the asymmetric 1H/1T stacking play a key role in suppressing superconductivity. Their potential role for shaping unconventional superconducting states, such as those reported in 6R- and 4H_b_-polytypes, warrants further investigation.

Overall, *in-situ* phase engineering in TMDs represents a powerful strategy for constructing hetero-phase superlattices with atomically sharp interfaces, controllable stacking sequences and tunable electronic states. Systematic exploration across TMD families will not only establish the generality of this approach, but also pave the way for designing metastable quantum architectures and realizing emergent phenomena in layered materials.

## MATERIALS AND METHODS

### Samples preparation

High-quality 1T-TaS_2_ and 2H-TaS_2_ single crystals were grown by a chemical vapor transport method, as described in note S1 [2]. The as-grown 1T-TaS_2_ crystals were transferred into a vacuum chamber (better than 10^−2^ mbar) and annealed at selected temperatures between 150 and 600°C for 3–4 h. The obtained crystals are classified according to annealing temperature as follows: LC-TaS_2_ (150–200°C), 1H_surf_-1T-TaS_2_ (200–250°C), 1H/multi-1T(i)-TaS_2_ (250–300°C), 1H/multi-1T(ii)-TaS_2_ (300–350°C), 1H/1T-TaS_2_ (350–400°C), 1H/1T/1H′(i)-TaS_2_ (450–500°C), 1H/1T/1H′(ii)-TaS_2_ (500–550°C), and 1H/1T/1H′(iii)-TaS_2_ (550–600°C). All crystals were cooled naturally to room temperature and stored in an Ar-protected glove box before further characterization. Subsequently, the crystals were cut to pieces for electron microscopy, synchrotron-based PES, and transport measurements.

### Electron microscopy

Cross-sectional STEM samples were cut from crystals using a focused ion beam system equipped with a gallium (Ga)-ion source. HAADF-STEM images were collected at room temperature on an FEI Themis Z aberration-corrected scanning transmission electron microscope operated at 300 kV with a convergence angle of 30 mrad.

### Synchrotron-based PES

High-resolution PES was collected at the BL11U beamline of the National Synchrotron Radiation Laboratory (NSRL, China). All crystals, except for the 1H_surf_(iii)-1T-TaS_2_ exhibiting a surface-limited phase transition, were cleaved in air and subsequently transferred into an ultra-high vacuum chamber with a base pressure of 1 × 10^−10^ mbar. The S 2*p* and Ta 4*f* spectra were measured at normal emission and room temperature using 240 eV photon energy. The photon energy was calibrated using the Au 4*f*_7/2_ core-level peak (84.0 eV) of a gold foil electrically contacted with the samples. The least-squares peak fitting was performed employing a Shirley background and asymmetric peak profiles for TaS_2_ species.

### Transport measurement

The electrical transport measurements for a series of freshly cleaved phase-engineered TaS_2_ crystals (note that cleavage was not performed on the four 1H_surf_(i/ii/iii/iv)-1T-TaS_2_ samples) were carried out by using a physical property measurement system (PPMS, Quantum Design) with a standard six-probe method. The warming and cooling rates are 3 K/min. Figure S14 shows an optical image of a bulk crystal for transport measurement, along with a schematic illustration of the electrode configuration.

### DFT calculation

The first-principles calculations based on DFT were implemented using the projector augmented-wave method [53] and the generalized gradient approximation within the Vienna Ab-initio Simulation Package [54]. The Perdew–Burke–Ernzerhof functional was used as the electron exchange-correlation functional [55]. In all calculations, the energy cutoff for the plane wave was set to 400 eV. Additionally, in the supercell or slab calculations, a vacuum layer of 20 Å was employed to avoid out-of-plane electronic and dipole–dipole interactions between adjacent layers. The vdW interactions were included by using the optB86b functional. The *k*-point interval in the first Brillouin zone was set to 0.02 Å^−1^. Initially, the inter-layer distance was fixed at 5.87 Å, corresponding to that of pristine 1T-TaS_2_ crystals. Structural optimization along the out-of-plane direction was then performed. The detailed computational workflow and validation tests are provided in note S5.

## Supplementary Material

nwag246_Supplemental_File

## References

[1] Manzeli S, Ovchinnikov D, Pasquier D et al. 2D transition metal dichalcogenides. Nat Rev Mater 2017; 2: 17033. 10.1038/natrevmats.2017.33

[2] Wang Y, Li Z, Luo X et al. Dualistic insulator states in 1T-TaS_2_ crystals. Nat Commun 2024; 15: 3425. 10.1038/s41467-024-47728-0 38653984 PMC11039681

[3] Tian N, Huang Z, Jang BG et al. Dimensionality-driven metal to Mott insulator transition in two-dimensional 1T-TaSe_2_. Natl Sci Rev 2023; 11: nwad144. 10.1093/nsr/nwad144 39678039 PMC11640825

[4] Darancet P, Millis AJ, Marianetti CA. Three-dimensional metallic and two-dimensional insulating behavior in octahedral tantalum dichalcogenides. Phys Rev B 2014; 90: 045134. 10.1103/PhysRevB.90.045134

[5] Li C-K, Yao X-P, Liu J et al. Fractionalization on the surface: is type-II terminated 1T-TaS_2_ surface an anomalously realized spin liquid? Phys Rev Lett 2022; 129: 017202. 10.1103/PhysRevLett.129.017202 35841554

[6] De la Barrera SC, Sinko MR, Gopalan DP et al. Tuning ising superconductivity with layer and spin-orbit coupling in two-dimensional transition-metal dichalcogenides. Nat Commun 2018; 9: 1427. 10.1038/s41467-018-03888-4 29650994 PMC5897486

[7] Vaňo V, Ganguli SC, Amini M et al. Evidence of nodal superconductivity in monolayer 1H-TaS_2_ with hidden order fluctuations. Adv Mater 2023; 35: 2305409. 10.1002/adma.202305409 37592888

[8] Geim AK, Grigorieva IV. Van der Waals heterostructures. Nature 2013; 499: 419–25. 10.1038/nature12385 23887427

[9] Ayani CG, Bosnar M, Calleja F et al. Unveiling the interlayer interaction in a 1H/1T TaS_2_ van der Waals heterostructure. Nano Lett 2024; 24: 10805–12. 10.1021/acs.nanolett.4c02068 39038223

[10] Vaňo V, Amini M, Ganguli SC et al. Artificial heavy fermions in a van der Waals heterostructure. Nature 2021; 599: 582–6. 10.1038/s41586-021-04021-0 34819682

[11] Liu M, Leveillee J, Lu S et al. Monolayer 1T-NbSe_2_ as a 2D-correlated magnetic insulator. Sci Adv 2021; 7: eabi6339. 10.1126/sciadv.abi6339 34797708 PMC8604411

[12] Ruan W, Chen Y, Tang S et al. Evidence for quantum spin liquid behaviour in single-layer 1T-TaSe_2_ from scanning tunnelling microscopy. Nat Phys 2021; 17: 1154–61. 10.1038/s41567-021-01321-0

[13] Persky E, Bjørlig AV, Feldman I et al. Magnetic memory and spontaneous vortices in a van der Waals superconductor. Nature 2022; 607: 692–6. 10.1038/s41586-022-04855-2 35896649

[14] Yan L, Ding C, Li M et al. Modulating charge-density wave order and superconductivity from two alternative stacked monolayers in a bulk 4H_b_-TaSe_2_ heterostructure via pressure. Nano Lett 2023; 23: 2121–8. 10.1021/acs.nanolett.2c04385 36877932

[15] Achari A, Bekaert J, Sreepal V et al. Alternating superconducting and charge density wave monolayers within bulk 6R-TaS_2_. Nano Lett 2022; 22: 6268–75. 10.1021/acs.nanolett.2c01851 35857927 PMC9373026

[16] Liu S-B, Tian C, Fang Y et al. Nematic ising superconductivity with hidden magnetism in few-layer 6R-TaS_2_. Nat Commun 2024; 15: 7569. 10.1038/s41467-024-51631-z 39217153 PMC11365993

[17] Yan L, Bu K, Li Z et al. Double superconducting dome of quasi two-dimensional TaS_2_ in non-centrosymmetric van der Waals heterostructure. Nano Lett 2024; 24: 6002–9. 10.1021/acs.nanolett.4c00579 38739273

[18] Wang S, Han Y, Sun S et al. Pressure induced nonmonotonic evolution of superconductivity in 6R-TaS_2_ with a natural bulk van der Waals heterostructure. Phys Rev Lett 2024; 133: 056001. 10.1103/PhysRevLett.133.056001 39159112

[19] Luo C, Cao G, Wang B et al. Self-assembly of 1T/1H superlattices in transition metal dichalcogenides. Nat Commun 2024; 15: 10584. 10.1038/s41467-024-54948-x 39632862 PMC11618666

[20] Mahatha SK, Phillips J, Corral-Sertal J et al. Self-stacked 1T-1H layers in 6R-NbSeTe and the emergence of charge and magnetic correlations due to ligand disorder. ACS Nano 2024; 18: 21052–60. 10.1021/acsnano.4c02005 39086092

[21] Silber I, Mathimalar S, Mangel I et al. Two-component nematic superconductivity in 4H_b_-TaS_2_. Nat Commun 2024; 15: 824. 10.1038/s41467-024-45169-3 38280890 PMC10821864

[22] Ribak A, Skiff RM, Mograbi M et al. Chiral superconductivity in the alternate stacking compound 4H_b_-TaS_2_. Sci Adv 2020; 6: eaax9480. 10.1126/sciadv.aax9480 32258393 PMC7101217

[23] Almoalem A, Feldman I, Mangel I et al. The observation of π-shifts in the Little-Parks effect in 4H_b_-TaS_2_. Nat Commun 2024; 15: 4623. 10.1038/s41467-024-48260-x 38816364 PMC11139670

[24] Gao JJ, Si JG, Luo X et al. Origin of the large magnetoresistance in the candidate chiral superconductor 4H_b_-TaS_2_. Phys Rev B 2020; 102: 075138. 10.1103/PhysRevB.102.075138

[25] Yang FZ, Zhang HD, Mandal S et al. Signature of magnetoelectric coupling driven finite momentum pairing in 3D ising superconductor. Nat Commun 2025; 16: 6626. 10.1038/s41467-025-61882-z 40681507 PMC12274449

[26] Yan L, Gao J, Zhang Z et al. Enhanced ising superconductivity in a unicompositional bulk 4H_b_-TaS_2_ superlattice via pressure. npj Quantum Mater 2025; 10: 104. 10.1038/s41535-025-00823-x

[27] Wattamaniuk WJ, Tidman JP, Frindt RF. Tunneling conductivity in 4H_b_-TaS_2_. Phys Rev Lett 1975; 35: 62–5. 10.1103/PhysRevLett.35.62

[28] Wang H, Jiao Y, Meng F et al. Evidence for multiband gapless superconductivity in the topological superconductor candidate 4H_b_-TaS_2_. Phys Rev Lett 2025; 135: 126002. 10.1103/d13p-mtbz 41046423

[29] Wan Z, Qian Q, Huang Y et al. Layered hybrid superlattices as designable quantum solids. Nature 2024; 635: 49–60. 10.1038/s41586-024-07858-3 39506149

[30] Li J, Yang X, Zhang Z et al. Towards the scalable synthesis of two-dimensional heterostructures and superlattices beyond exfoliation and restacking. Nat Mater 2024; 23: 1326–38. 10.1038/s41563-024-01989-8 39227467

[31] Sung SH, Schnitzer N, Novakov S et al. Two-dimensional charge order stabilized in clean polytype heterostructures. Nat Commun 2022; 13: 413. 10.1038/s41467-021-27947-5 35058434 PMC8776735

[32] Husremović S, Goodge BH, Erodici MP et al. Encoding multistate charge order and chirality in endotaxial heterostructures. Nat Commun 2023; 14: 6031. 10.1038/s41467-023-41780-y 37758701 PMC10533556

[33] Li W, Qian X, Li J. Phase transitions in 2D materials. Nat Rev Mater 2021; 6: 829–46. 10.1038/s41578-021-00304-0

[34] Liu X, Shan J, Cao T et al. On-device phase engineering. Nat Mater 2024; 23: 1363–9. 10.1038/s41563-024-01888-y 38664497

[35] Lim J, Lee J-I, Wang Y et al. Photoredox phase engineering of transition metal dichalcogenides. Nature 2024; 633: 83–9. 10.1038/s41586-024-07872-5 39198653 PMC11374681

[36] Lin Y-C, Dumcenco DO, Huang Y-S et al. Atomic mechanism of the semiconducting-to-metallic phase transition in single-layered MoS_2_. Nat Nano 2014; 9: 391–6. 10.1038/nnano.2014.64 24747841

[37] Yin X, Wang Q, Cao L et al. Tunable inverted gap in monolayer quasi-metallic MoS_2_ induced by strong charge-lattice coupling. Nat Commun 2017; 8: 486. 10.1038/s41467-017-00640-2 28883392 PMC5589873

[38] Wang Z, Sun Y-Y, Abdelwahab I et al. Surface-limited superconducting phase transition on 1T-TaS_2_. ACS Nano 2018; 12: 12619–28. 10.1021/acsnano.8b07379 30403840

[39] Wu S, Tian W, Li R et al. Self-intercalated 6R-TaS_2_ with reduced symmetry for room temperature nonlinear hall effect. Matter 2025; 8: 102153. 10.1016/j.matt.2025.102153

[40] Lazar P, Martincová J, Otyepka M. Structure, dynamical stability, and electronic properties of phases in TaS_2_ from a high-level quantum mechanical calculation. Phys Rev B 2015; 92: 224104. 10.1103/PhysRevB.92.224104

[41] Guo Y, Sun D, Ouyang B et al. Probing the dynamics of the metallic-to-semiconducting structural phase transformation in MoS_2_ crystals. Nano Lett 2015; 15: 5081–8. 10.1021/acs.nanolett.5b01196 26134736

[42] Voiry D, Mohite A, Chhowalla M. Phase engineering of transition metal dichalcogenides. Chem Soc Rev 2015; 44: 2702–12. 10.1039/C5CS00151J 25891172

[43] Hughes HP, Starnberg HI. Electron Spectroscopies Applied to Low-dimensional Materials. New York: Springer, 2001.

[44] Bae H, Valentí R, Mazin II et al. Designing flat bands, localized and itinerant states in TaS_2_ trilayer heterostructures. npj Quantum Mater 2025; 10: 92. 10.1038/s41535-025-00812-0

[45] Ritschel T, Trinckauf J, Koepernik K et al. Orbital textures and charge density waves in transition metal dichalcogenides. Nat Phys 2015; 11: 328–31. 10.1038/nphys3267

[46] Shao DF, Xiao RC, Lu WJ et al. Manipulating charge density waves in 1T-TaS_2_ by charge-carrier doping: a first-principles investigation. Phys Rev B 2016; 94: 125126. 10.1103/PhysRevB.94.125126

[47] Almoalem A, Gofman R, Nitzav Y et al. Charge transfer and spin-valley locking in 4H_b_-TaS_2_. npj Quantum Mater 2024; 9: 36. 10.1038/s41535-024-00646-2

[48] Yang Y, Fang S, Fatemi V et al. Enhanced superconductivity upon weakening of charge density wave transport in 2H-TaS_2_ in the two-dimensional limit. Phys Rev B 2018; 98: 035203. 10.1103/PhysRevB.98.035203

[49] Inada R, Ōnuki Y, Tanuma S. Hall effect of 1T-TaS_2_. Phys Lett A 1979; 69: 453–6. 10.1016/0375-9601(79)90406-7

[50] Naito M, Tanaka S. Electrical transport properties in 2H-NbS_2_, -NbSe_2_, -TaS_2_ and -TaSe_2_. J Phys Soc Jpn 1982; 51: 219–27. 10.1143/JPSJ.51.219

[51] Peng J, Yu Z, Wu J et al. Disorder enhanced superconductivity toward TaS_2_ monolayer. ACS Nano 2018; 12: 9461–6. 10.1021/acsnano.8b04718 30126279

[52] Liu C-X . Unconventional superconductivity in bilayer transition metal dichalcogenides. Phys Rev Lett 2017; 118: 087001. 10.1103/PhysRevLett.118.087001 28282184

[53] Blöchl PE . Projector augmented-wave method. Phys Rev B 1994; 50: 17953–79. 10.1103/PhysRevB.50.17953 9976227

[54] Kresse G, Hafner J. Ab initio molecular dynamics for liquid metals. Phys Rev B 1993; 47: 558–61. 10.1103/PhysRevB.47.558 10004490

[55] Perdew JP, Burke K, Ernzerhof M. Generalized gradient approximation made simple. Phys Rev Lett 1996; 77: 3865–8. 10.1103/PhysRevLett.77.3865 10062328

