# Supplementary material for: Self-adaptive hetero-phase superlattices in TaS2 via layer-resolved 1T-to-1H transformations
Source: Natl Sci Rev. 2026 Apr 28;13(11):nwag246. doi: 10.1093/nsr/nwag246 (PMC13281099; doi:10.1093/nsr/nwag246)
Supplement: nwag246_Supplemental_File [file nwag246_supplemental_file.pdf]

Supporting Information

Self-adaptive hetero-phase superlattices in TaS_2 via layer-resolved 1T-to-1H transformations

Zhenyu Ding,[1,2,3,†] Yihao Wang,[1,†] Rui Li,[1,3] Jingjing Gao,[4] Jialiang Jiang,[1] Jin Tang,[5,6] Yuyan Han,[1] Qian Xu,[7] Junfa Zhu,[7] Wenqian Tu,[4] Wenjian Lu,[4] Yingguo Yang,[8] Zhihao Li,[1,9] Xingyu Gao,[10] Zhe Qu,[1] Yuping Sun,[1,4,11] Xuan Luo,[4,*] Xiaoping Yang,[1,*] Hai Xu,[2,12,*] Yimin Xiong,[5,6,13,*] Liang Cao[1,*]

[1]Anhui Provincial Key Laboratory of Low-Energy Quantum Materials and Devices, High Magnetic Field Laboratory, HFIPS, Chinese Academy of Sciences, Hefei 230031, China;

[2]School of Optoelectronic Science and Engineering, Anhui University, Hefei 230601, China;

[3]Science Island Branch of Graduate School, University of Science and Technology of China, Hefei 230026, China;

[4]Key Laboratory of Materials Physics, Institute of Solid State Physics, HFIPS, Chinese Academy of Sciences, Hefei 230031, China;

[5]Department of Physics, School of Physics, Anhui University, Hefei 230601, China;

[6]Anhui Provincial Key Laboratory of Magnetic Functional Materials and Devices, Anhui University, Hefei 230601, China;

[7]National Synchrotron Radiation Laboratory, University of Science and Technology of China, Hefei 230026, China;

[8]State Key Laboratory of Photovoltaic Science and Technology, School of Microelectronics, Fudan University, Shanghai 200433, China;

[9]Yangtze Memory Technologies Co., Ltd., Wuhan 430205, China;

[10]Shanghai Synchrotron Radiation Facility (SSRF), Zhangjiang Laboratory, Shanghai Advanced Research Institute, Chinese Academy of Sciences, Shanghai 201204, China;

[11]Collaborative Innovation Center of Advanced Microstructures, Nanjing University, Nanjing 210093, China;

[12]State Key Laboratory of Opto-Electronic Information Acquisition and Protection Technology, Anhui University, Hefei 230601, China;

[13]Hefei National Laboratory, Hefei 230028, China

Supplementary Note 1. Growth of 1T-TaS_2 and 2H-TaS_2 single crystals

High-quality 1T-TaS_2 single crystals were grown by a chemical vapor transport (CVT) method [1-4]. First, tantalum and sulfur powder in a 1:2 molar ratio were mixed and sealed under vacuum in quartz tubes, and annealed at 800°C for 3 days. The resulting polycrystalline TaS_2 was quenched in an ice-water mixture. Subsequently, the polycrystalline TaS_2 powder and iodine (as a transport agent) were vacuum-sealed in quartz tubes. The quartz tubes were heated in a two-zone furnace for 10 days with hot and cold zones maintained at 850°C and 750°C, respectively. After quenching, the 1T-TaS_2 crystals were obtained, whereas slow cooling to room temperature yielded 2H-TaS_2 crystals.

Supplementary Note 2. DFT calculated band structure of TaS_2 atomic-layers

For the band structure simulation, the √13×√13 and 3×3 supercells incorporating CDW-induced distortions were considered for 1T-TaS_2 and 1H-TaS_2 atomic-layers, respectively. The on-site Coulomb repulsion U=2.94 eV, previously established using a self-consistent method [5], was included for the Ta 5*d* orbitals. The Brillouin zone was sampled on the Monkhorst-Pack *k*-points with a mesh of 9×9×1 for the self-consistent calculation.

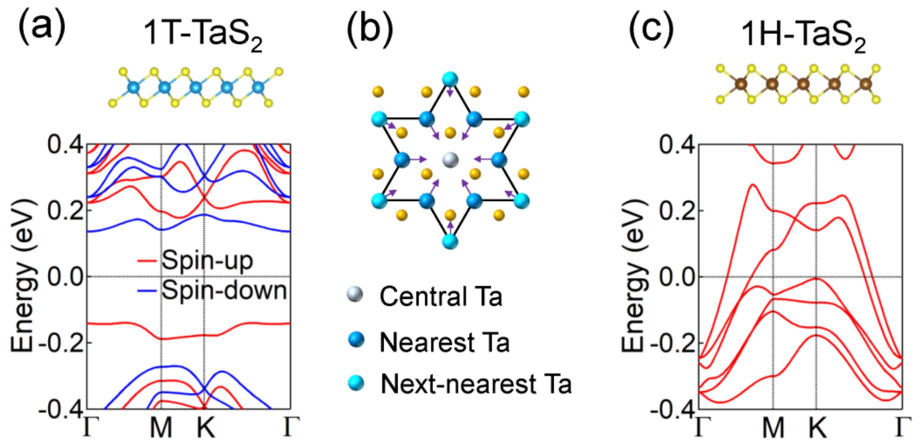

Figure S1. Distinct electronic band structures of 1T- and 1H-TaS_2 atomic-layer. (a) Calculated band structure of 1T-TaS_2 atomic-layer in the well-known √13×√13 CDW states. (b) Atomic configuration of the corresponding CDW David-star cluster, where 12 outer Ta-atoms are displaced inward the central Ta-atom. (c) Calculated band structure of 1H-TaS_2 atomic-layer in the 3×3 CDW states.

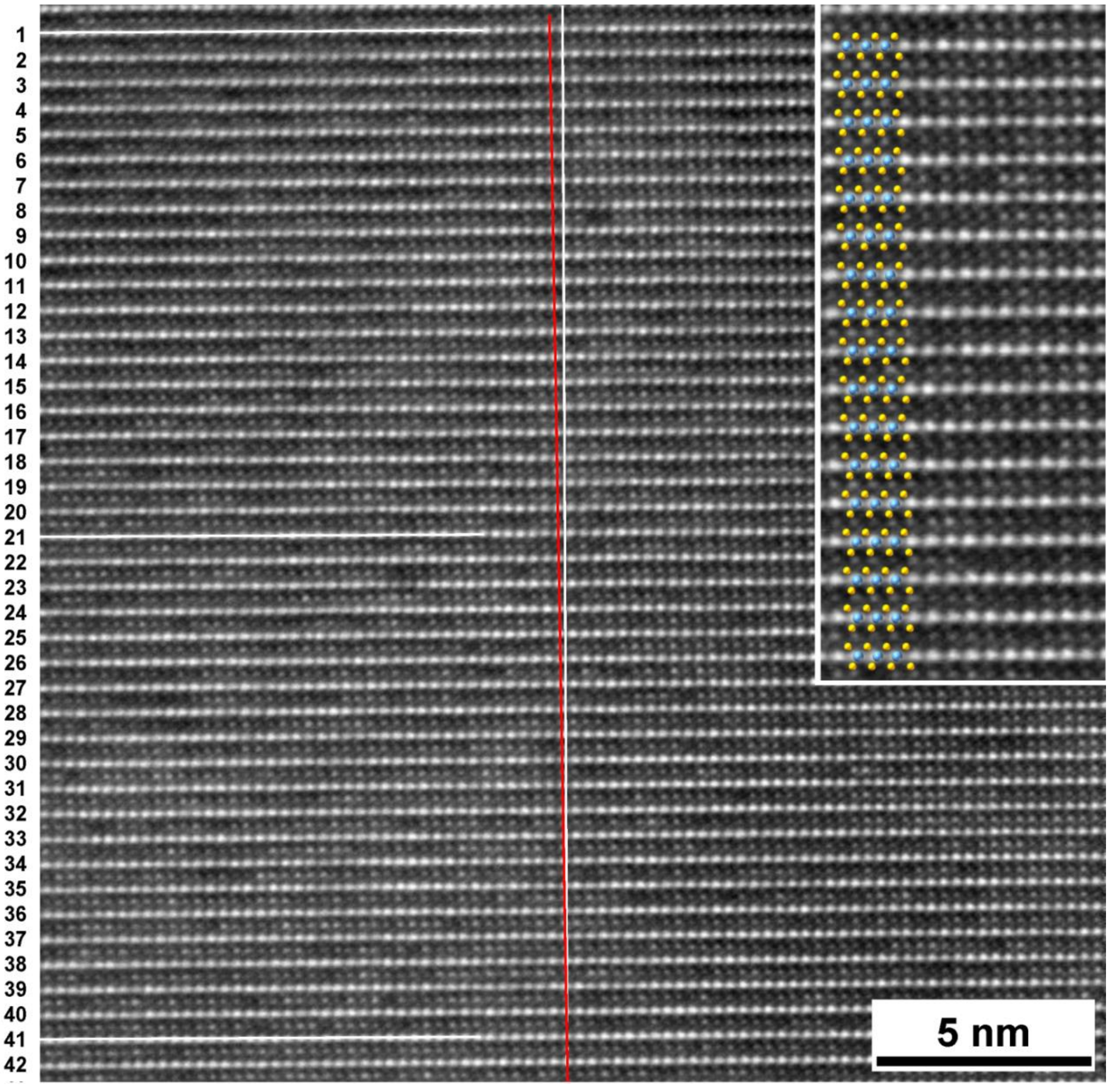

Figure S2. Collective inter-layer sliding in LC-TaS_2 crystals. The cross-sectional HAADF-STEM images of LC-TaS_2 crystals collected along the [110] direction. The vertical white line indicates the out-of-plane *c*-axis direction, and red line highlights the inter-layer misalignment.

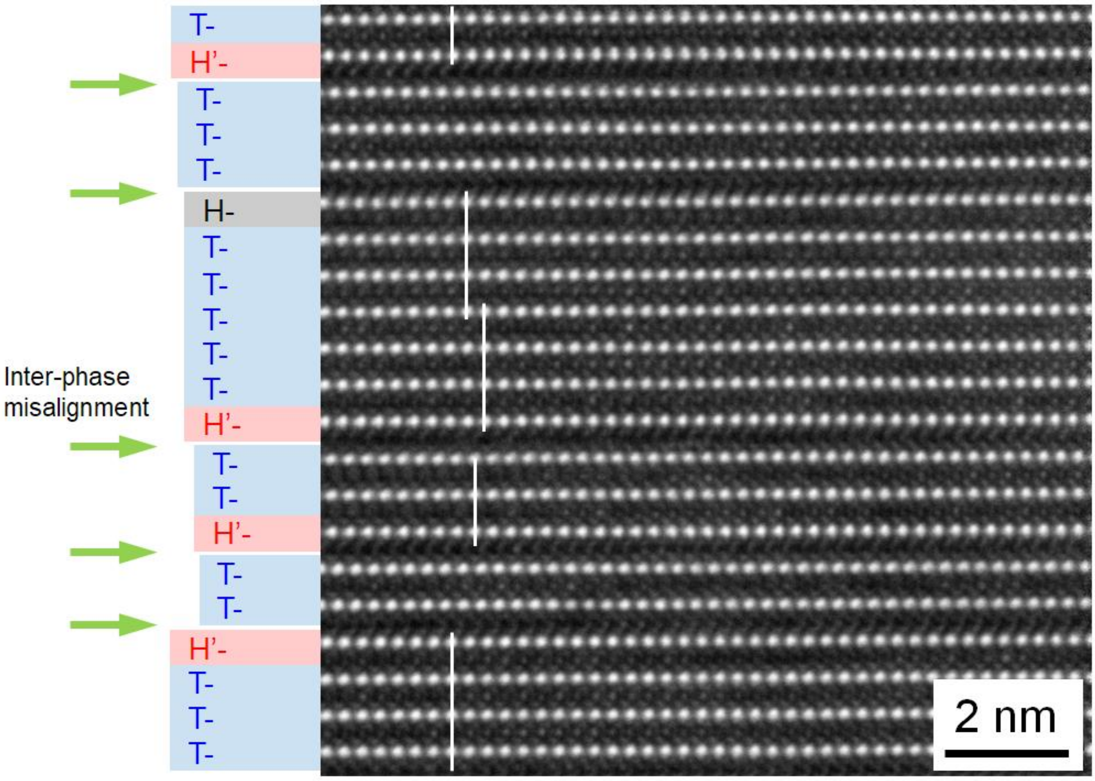

Figure S3. The internal individual-layer 1T-to-(1H or 1H') transition and the resulting 1H/multilayer-1T superlattice in 1H/multi-1T(i)-TaS_2 crystals. The cross-sectional HAADF-STEM images of 1H/multi-1T(i)-TaS_2 crystals collected along the [100] direction. The white lines highlight the 1H/multilayer-1T or multilayer-1T/1H' units characterized by restored vertical Ta-Ta alignment.

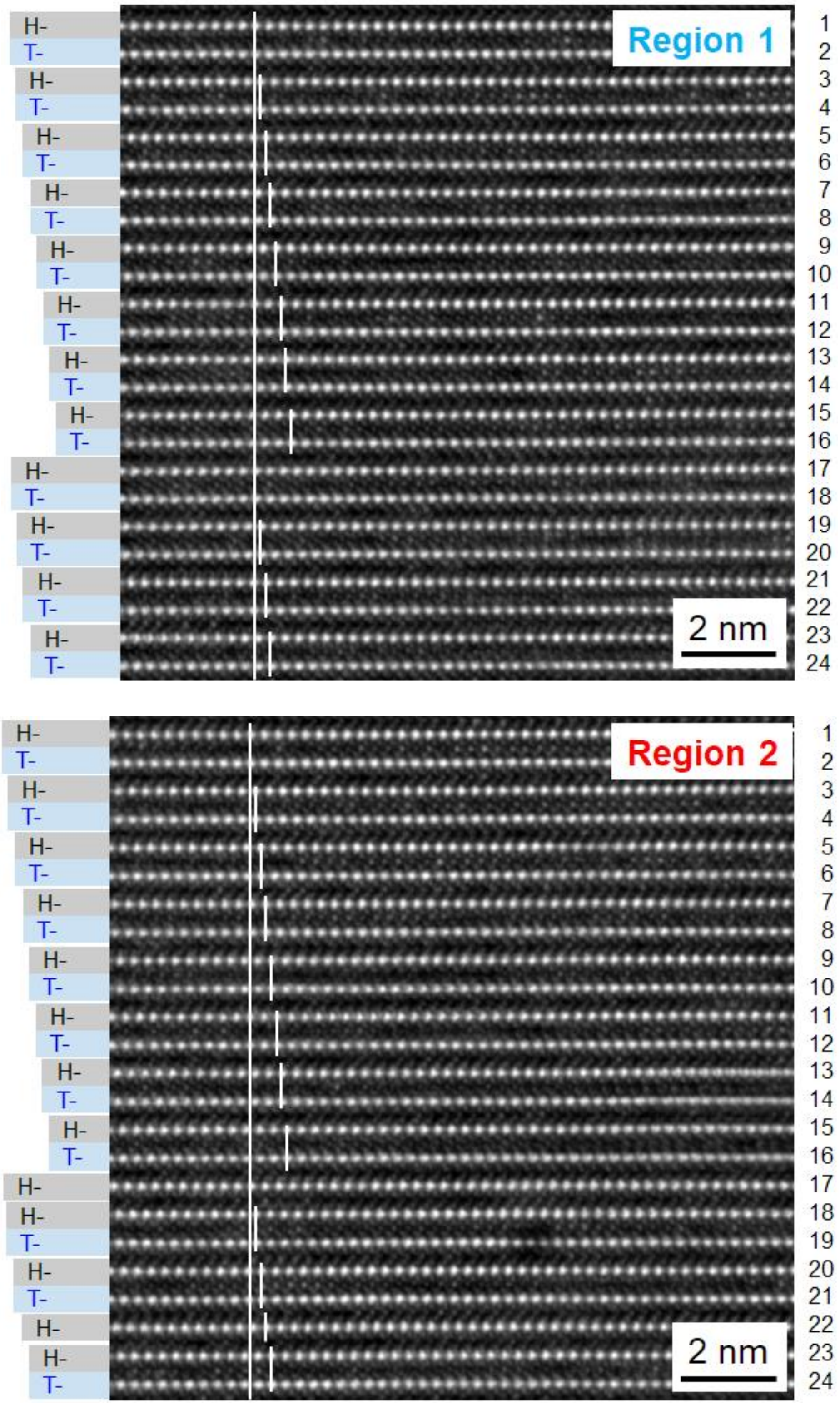

Figure S4. Paired 1H/1T superlattice in 1H/1T-TaS_2 crystals. The cross-sectional HAADF-STEM images along the [100] direction collected at two regions of the 1H/1T-TaS_2 crystal, separated by millimeter-scale distances.

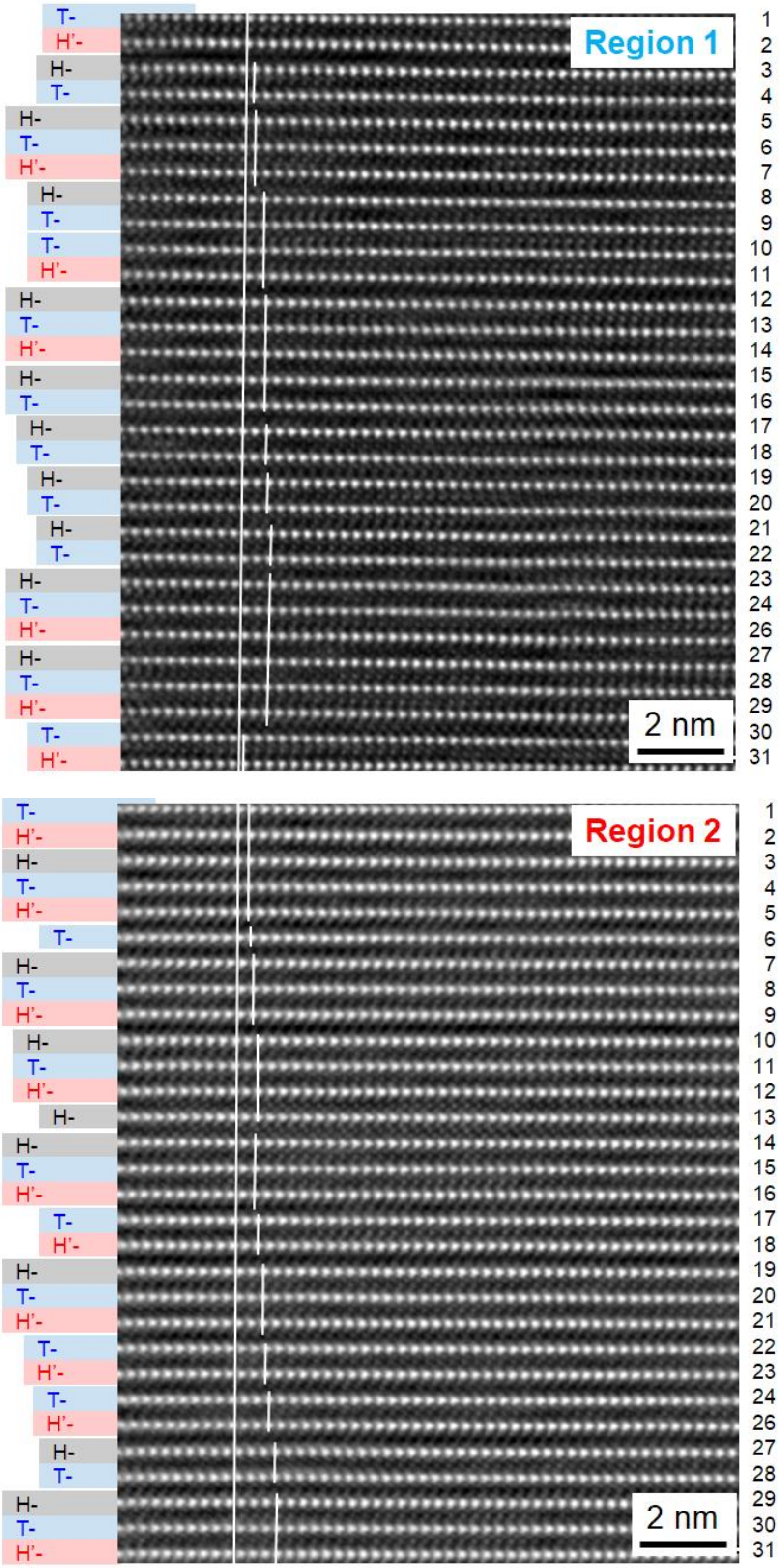

Figure S5. Sandwiched 1H/1T/1H' superlattice units in 1H/1T/1H'(iii)-TaS_2 crystals. The cross-sectional HAADF-STEM images along the [100] direction collected at two regions of 1H/1T/1H'(iii)-TaS_2 crystal, separated by millimeter-scale distances.

To verify the phase configurations of the hetero-phase superlattices, Raman measurements were performed at 22 K and shown in Fig. S6. These data further support the formation of well-defined hetero-phase superlattice structures.

At 22 K, 1T-TaS_2 exhibits multiple Raman modes associated with commensurate charge density wave (C-CDW), whereas 2H-TaS_2 shows a dominant A_{1g} mode at ~400 cm^{-1}, consistent with previous reports [6, 7]. In the 1H/multi-1T(i)-TaS_2, these 1T-TaS_2 associated modes show a noticeable blue shift, indicating hardening of the C-CDW related vibration. Upon the formation of the paired 1H/1T superlattice, most C-CDW modes are suppressed, and the A_{1g} mode of the 1H layer becomes dominant, with a blue shift relative to 2H-TaS_2. The hardening and suppression of the C-CDW related vibrational modes, together with the blue shift of A_{1g} mode, indicates electron transfer from the 1T to 1H layers at lower temperature, consistent with synchrotron-based photoemission spectroscopy (PES) results.

In addition to the A_{1g} mode of 1H component, additional modes ~160 cm^{-1} and ~280 cm^{-1} are observed. Specifically, the ~160 cm^{-1} mode is attributed to the CDW-related vibrations of the 1H layer [8]. The origin of the ~280 cm^{-1} mode, whether associated with the 1H- or 1T-TaS_2 layer component, is unclear. In the 1H/1T/1H' superlattice, these modes remain almost unchanged, whereas the A_{1g} mode undergoes a further blue shift, indicating enhanced electron transfer to 1H or 1H' layers. Although Raman mode of the 1T component are not well resolved, mostly due to strong electronic screening, the presence of 1H related modes and systematic A_{1g} shift enable clear distinction between 1H/1T and 1H/1T/1H' superlattice configurations.

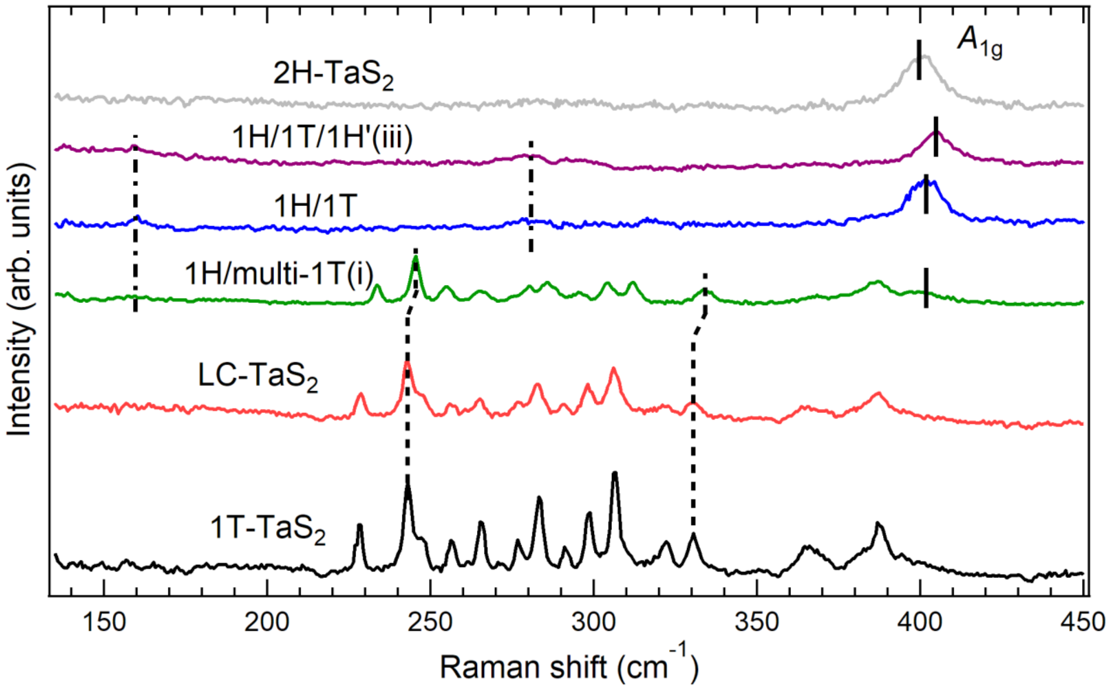

Figure S6. Raman spectra for 1T-, LC-, 1H/multi-1T(i)-, 1H/1T-, 1H/1T/1H'(iii)- and $2H\text{-}TaS_2$ crystals collected at 22 K.

Supplementary Note 3: The bright spots in the van der Waals gaps of scanning transmission electron microscopy (STEM) images (Fig. 1g–l) are attributed to Ga-atoms unintentionally introduced during FIB processing for STEM sample preparation, rather than self-intercalated Ta-atoms

The reasons are summarized below.

(i) Negligible self-intercalated Ta-atoms in pristine 1T-TaS_2 crystals.
Synchrotron-based high-resolution Ta 4*f* spectra (Fig. 3b in the main text) show no evidence of self-intercalated Ta. In contrast, in our previous study on Ti self-intercalation in TiS_2, self-intercalated Ti-atoms produced a characteristic lower binding energy shoulder in the Ti 2*p* spectrum due to photo-hole screening effect [9]. No such feature is observed in Ta 4*f* spectra, indicating that possible self-intercalation is below the detection limits (<1 at%).

(ii) Absence of foreign atomic intercalation in pristine 1T-TaS_2 crystals.
Lab-based X-ray photoemission spectroscopy (XPS, Fig. S7a) collected from five air-cleaved 1T-TaS_2 crystals reveals only the characteristic Ta- and S-signals, together with minor containment C- and O-signals, indicating the absence of foreign elements in the crystals. The Ta:S ratio, determined from Ta 4*f* and S 2*p* core level spectra (Fig. S7b and c), is 0.96:2, closely matching the expected stoichiometry of 1:2.

(iii) Stoichiometry preserved after annealing. The Ta:S ratio remains almost constant for all crystals, as verified by synchrotron-based Ta 4*f* and S 2*p* core level spectra (Fig. S8), indicating that no thermally induced intercalation or stoichiometric deviation occurs during the annealing process.

(iv) Presence of Ga-artifact in STEM samples. Energy-dispersive X-ray spectroscopy (EDX) spectrum and elemental mapping (Fig. S9) obtained from the STEM sample reveal the presence of Ta, S, and additional Ga-signals peaked at ~1 and ~9 keV. Specifically, Ga-ion bombardment during FIB milling result in partial implantation of Ga-atoms into the vdW gaps, giving rise to the observed bright features.

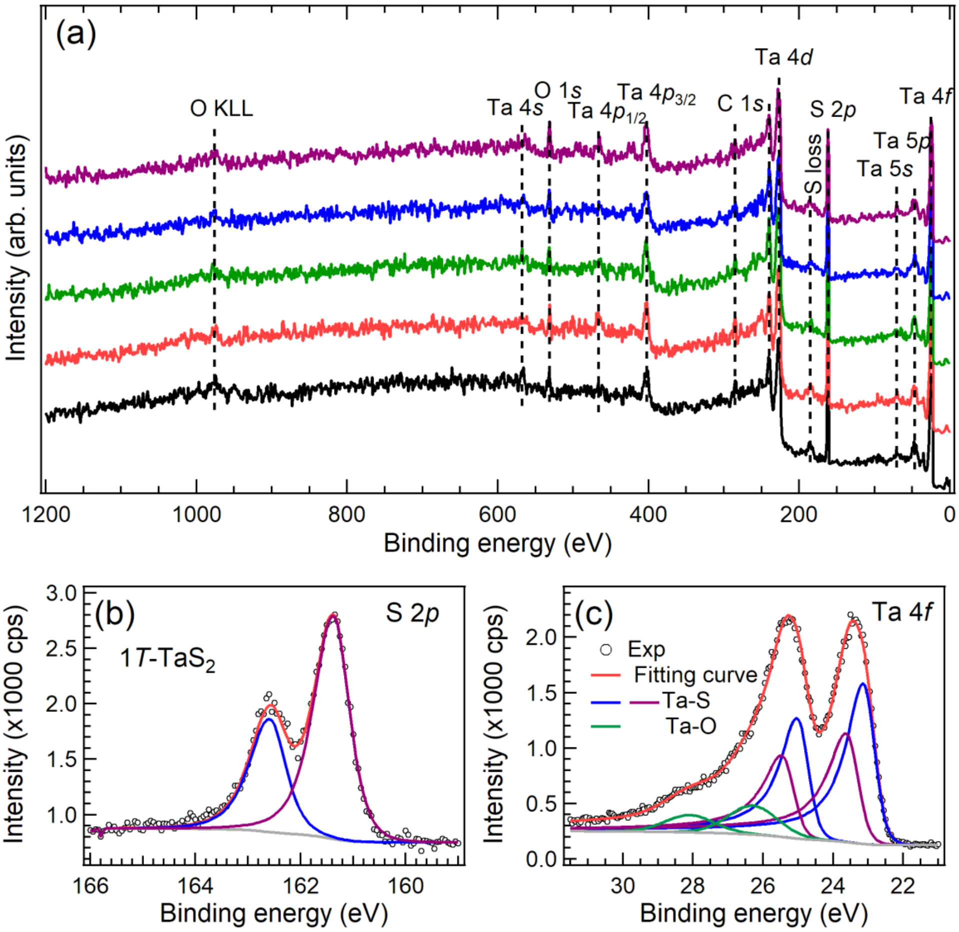

Figure S7. XPS characterization of air-cleaved 1T-TaS_2 crystals. (a) The XPS survey spectra of five air-cleaved 1T-TaS_2 crystals measured by Al *K*α X-ray source (1486.6 eV), showing characteristic Ta and S signals along with minor C and O peak, indicative of no foreign elements. (b) S 2*p* and (c) Ta 4*f* core level spectra of a representative 1T-TaS_2 crystal. The Ta:S atomic ratio of 0.96:2, derived from integrated area of the Ta-S bonding features, closely matches the expected stoichiometric ratio, confirming the compositional integrity of the crystals.

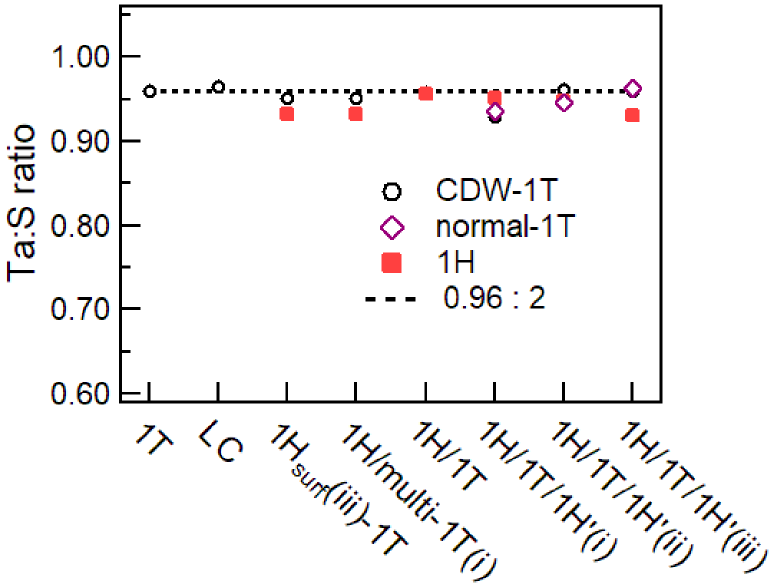

Figure S8. Atomic ratio determined from synchrotron-based photoemission spectroscopy (PES). The elemental Ta:S ratio for all three species, derived from synchrotron-based Ta 4*f* and S 2*p* core-level spectra (Fig. 3a and b in the main text). The horizontal dashed line represents the Ta:S ratio of 0.96:2 of pristine 1T-TaS_2 crystal yielded from lab-based XPS spectra, serving as a stoichiometric reference.

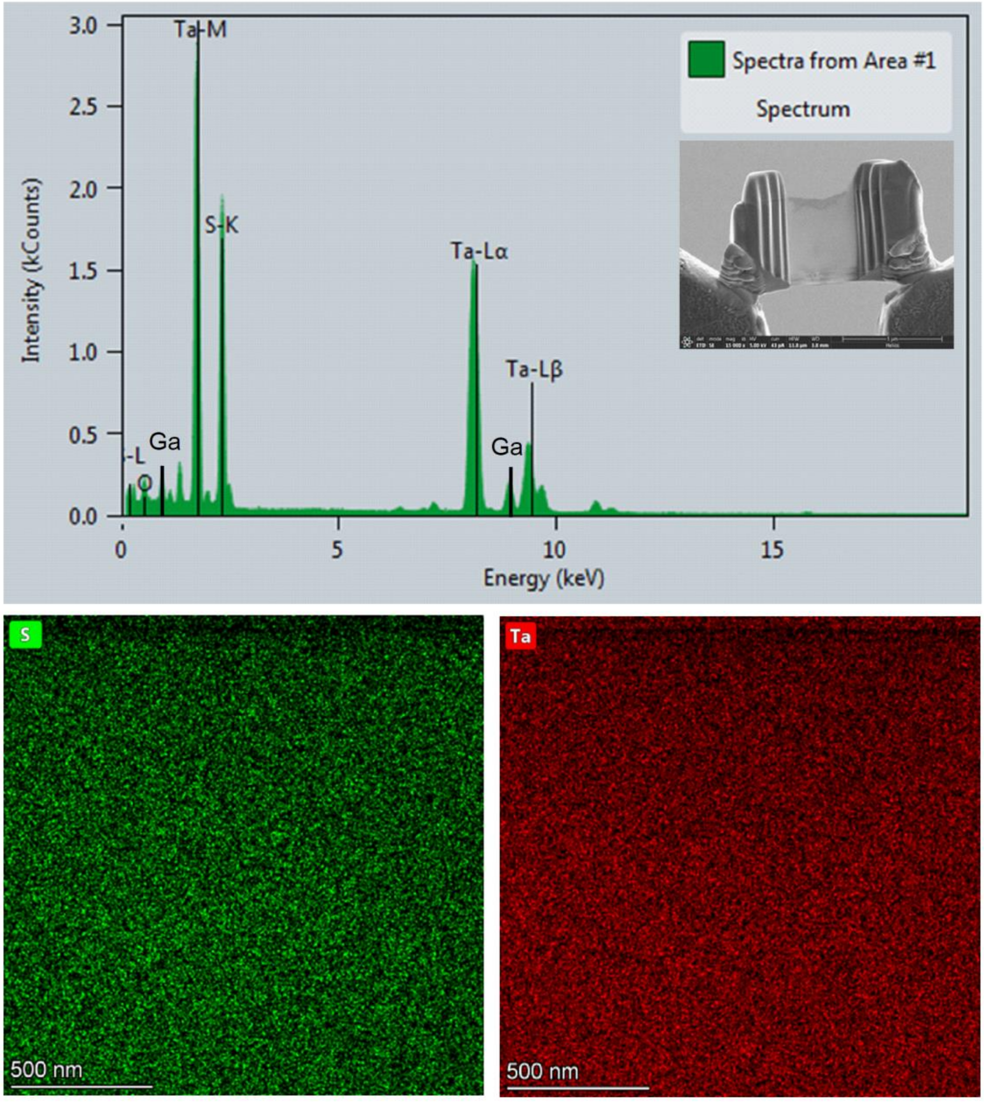

Figure S9. EDX and elemental mapping of the STEM sample. EDX spectrum of a STEM specimen (inset) cut from the pristine 1T-TaS_2 crystal. Cross-sectional STEM samples were cut from crystals using a focused ion beam system equipped with a gallium (Ga)-ion source. To minimize ion-beam damage during milling, a protective carbon layer was first deposited on the crystal surfaces, followed by a platinum (Pt) layer. The features close to 1 and 9 keV are attributed to Ga-element, unintentionally introduced during focused ion beam (FIB) processing. The bottom panels show the EDX elemental mapping of S and Ta, respectively.

Supplementary Note 4: Synchrotron-based X-ray diffraction (XRD) measurement

The synchrotron-based XRD measurements were performed at the BL14B1 beamline of the Shanghai Synchrotron Radiation Facility (SSRF) using X-rays with a wavelength of λ = 1.24 Å. The corresponding spectra, collected at room temperature, are presented in scattering vector q coordinates by using the equation $q=4\pi\sin\theta/\lambda$, where θ is half of the diffraction angle. The q has been calibrated by measuring the synchrotron-based XRD of a lanthanum hexaboride reference sample.

Grazing incident angles of 0.1° and 1.0° were employed to probe surface and bulk transformations. Notably, the peak at q=10.67 nm^{-1} in $1H_{surf}$(iii)-1T-TaS_2 appears solely at the surface sensitive angle of 0.1°, indicative of a surface-limited 1T-to-1H transition. The inter-layer distances extracted from the dominant diffraction peaks reveal the structural evolution in bulk regions.

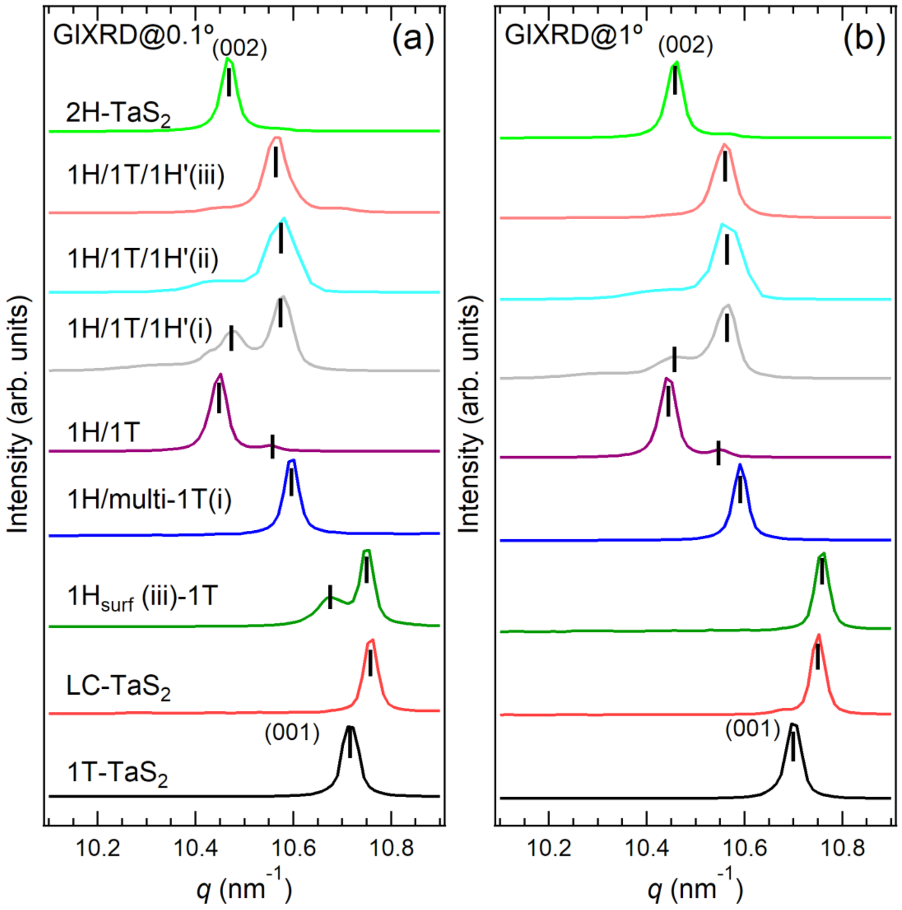

Figure S10. Surface 1T-to-1H transition and inter-layer distances determination. Synchrotron-based XRD spectra around the (001) diffraction peak collected at room temperature for a series of TaS_2 crystals. Notably, the peak at q=10.67 nm^{-1} in $1H_{surf}$(iii)-1T-TaS_2 appears at a surface sensitive incident angle of 0.1°, indicating a surface-limited 1T-to-1H transition. Inter-layer distances extracted from the dominant diffraction peaks reveal structural evolution in the bulk regions.

Supplementary Note 5: DFT calculated energetics

For numerical simulations in Fig. 2d of the main text, the energy evolution as a function of inter-layer or intra-layer S-plane displacement was calculated in a 1T-TaS_2 bilayer system. To validate intra-layer S-plane sliding direction, a **γ-**misaligned bilayer configuration was adopted, constraining translational motion of S-plane without swellings and buckling.

A set of total energy calculations in Fig. 2e of the main text were performed using a 6-layer slab system, simulating the structural transformation (Figs. 2a–c in the main text). The vdW interactions were included by using the optB86b functional. The *k*-point interval in the first Brillouin zone was set to 0.02 $Å^{-1}$. Initially, the inter-layer distance was fixed at 5.87 Å, corresponding to that of pristine 1T-TaS_2 crystals. Structural optimization along the out-of-plane direction was then performed. The DFT-relaxed average inter-layer distances show excellent agreement with experimental values determined from synchrotron-based XRD (Fig. S10 and Table S1), validating the reliability of structural models. In addition, after structural optimization, the calculated inter-layer distance for $4H_b$- and 2H-TaS_2 were 5.90 and 6.05 Å, respectively, in good agreement with reported experimental structures [10, 11].

To verify that the 6-layer slab model sufficiently captures the inter-layer coupling characteristic of bulk crystals, additional calculations were conducted on 12-layer slabs. As shown in Fig. S13a and b, the energy profiles for all stacking configurations remain nearly identical between the 6- and 12-layer models, indicating that the 6-layer slab system is sufficient to represent the essential inter-layer coupling.

Table S1. Inter-layer distances (Å) obtained from DFT-calculations and synchrotron-based XRD measurements.

Stacking configurations	DFT-calculated average interlayer distances	Interlayer distances from XRD	Sample
I	5.8920	5.8656	1T-TaS_2
II	5.8920	5.8448	LC-TaS_2
III	5.9490	5.9320	1H/multi-1T(i)-TaS_2
IV	5.9317	——	
V	6.0317	——	
VI	6.0187	——	
VII	6.0113	6.0161	1H/1T-TaS_2
VIII	6.1056	——	
IX	5.9622	5.9500	1H/1T/1H'(iii)-TaS_2
X	5.8916	——	

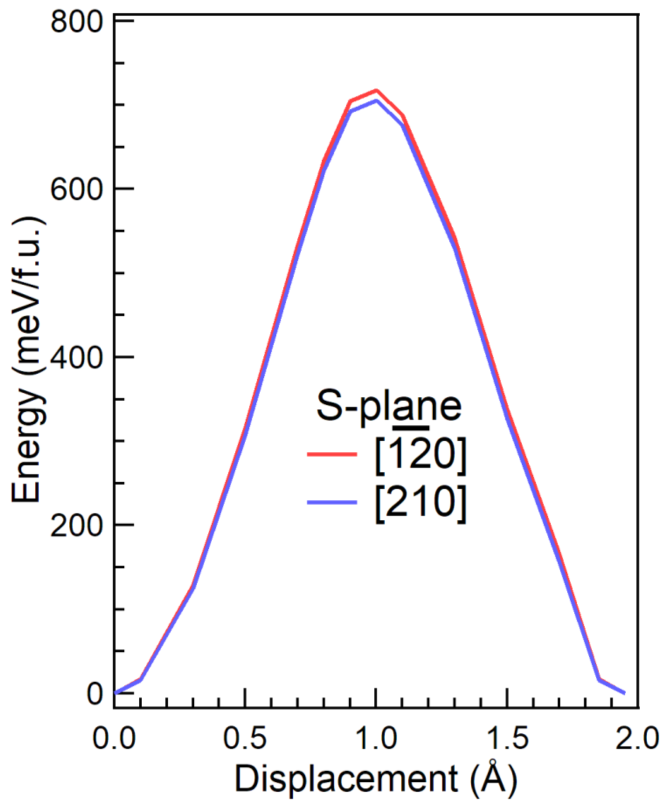

Figure S11. Energetically preferred [210] intra-layer S-plane sliding. Energy profiles for intra-layer S-plane sliding along [210] and [$\bar{1}\bar{2}0$] directions, as calculated from a 1T-TaS_2 bilayer system with a γ~0.15 Å inter-layer misalignment. The energy barrier along [210] (705.49 meV/f.u.) is slightly lower than that along [$\bar{1}\bar{2}0$] (717.35 meV/f.u.), indicating a preferred sliding direction.

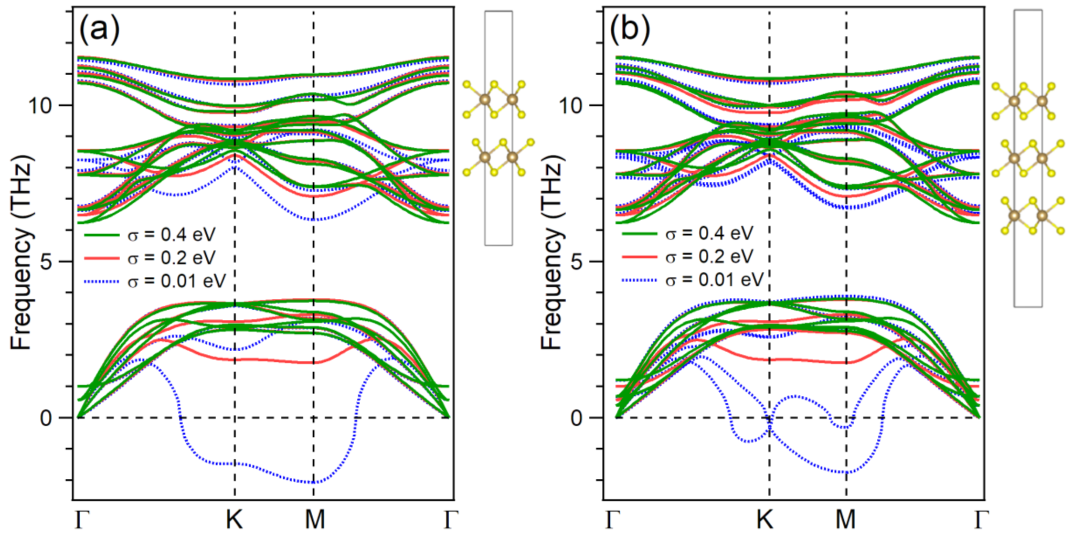

Figure S12. Phonon dispersion of hetero-phase TaS_2 superlattice units. Calculated phonon spectra for (a) the 1H/1T bilayer and (b) 1H/1T/1H' trilayer at electronic smearing values of σ= 0.01 eV, 0.2 eV, and 0.4 eV. Here, σ = 0.01 eV corresponds to low-temperature conditions, while σ= 0.2 eV and 0.4 eV represent elevated temperatures. Imaginary frequencies appear at σ= 0.01 eV, indicating dynamic instability at low temperatures. At higher smearing value, no imaginary modes are present. The softening of acoustic branches along the K-M path at σ= 0.2 eV disappears at σ= 0.4 eV. These results confirm that both 1H/1T and 1H/1T/1H' configurations are dynamically stable under annealing-relevant conditions.

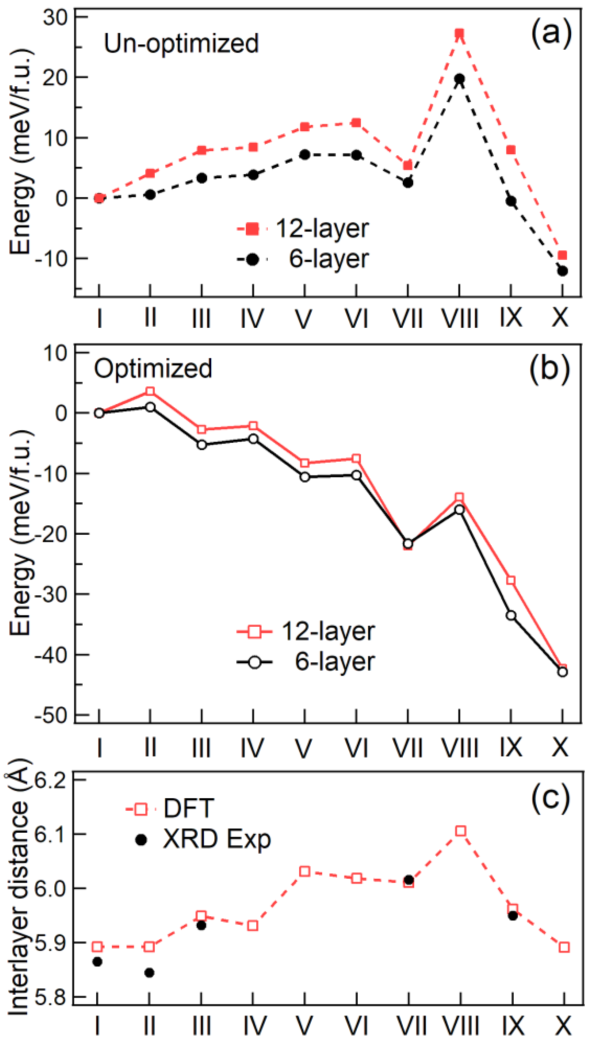

Figure S13. Layer thickness dependence of stacking energetics. The energy profiles for various stacking sequences (I-X in Fig. 2a–c of the main text) calculated using 6-layer and 12-layer slabs with (a) a fixed inter-layer spacing of 5.87 Å, and (b) optimized inter-layer spacing along *c*-axis. The near-identical trends indicate that the 6-layer model adequately captures inter-layer coupling and bulk behavior. (c) Averaged inter-layer distances obtained from DFT-calculations, in good agreement with experimental inter-layer distances determined by XRD (Fig. S10 and Table S1).

Supplementary Note 6: Electrical transport properties and superconductivity

The crystals were cut into rectangular pieces with typical dimensions of 2.0×1.5×0.25 mm^3. Platinum electrodes were attached using DuPont 4929N silver paint under ambient conditions, without encapsulation or protective coatings. The two current-electrodes were placed at opposite ends of crystals and covered with silver paint to ensure uniform longitudinal current across the bulk. The two voltage-electrodes were placed along the current direction, while the two Hall-electrodes were symmetrically positioned perpendicular to the current path.

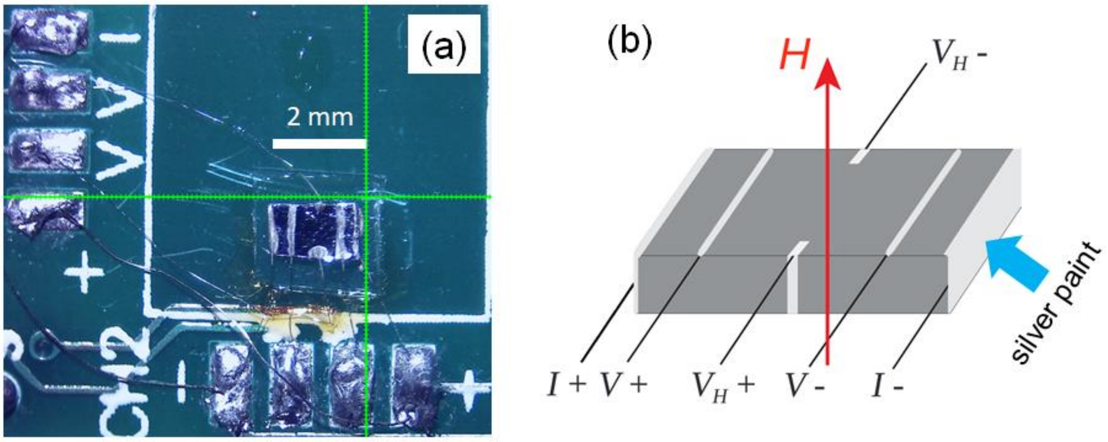

Figure S14. Bulk crystal transport measurement setup. (a) The optical image of a bulk crystal with a standard six-electrode setup for transport measurements. (b) Schematic drawing of the contact configuration for in-plane resistivity ρ_{ab} and Hall resistivity ρ_{xy} measurements.

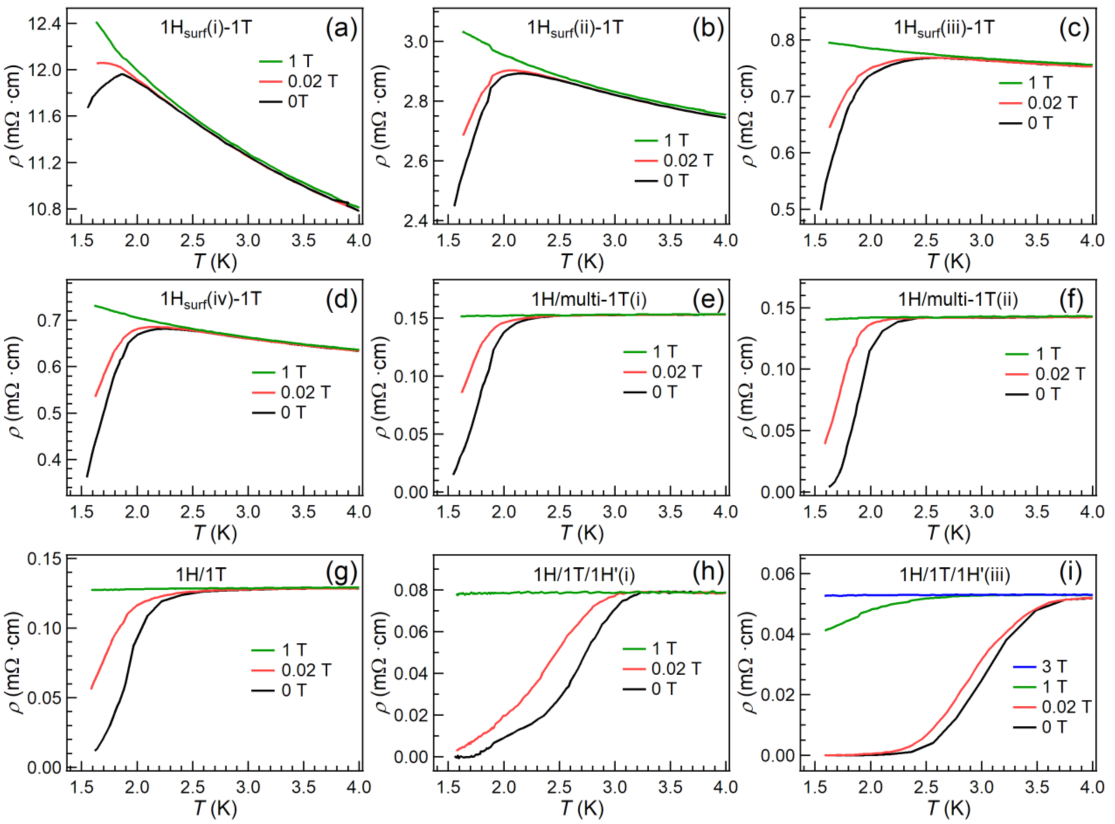

Figure S15. Magnetic-field-dependent resistivity near superconducting transition. Temperature-dependent in-plane resistivity of treated TaS_2 crystals measured under varying applied magnetic field, highlighting the suppression of superconductivity with increasing field.

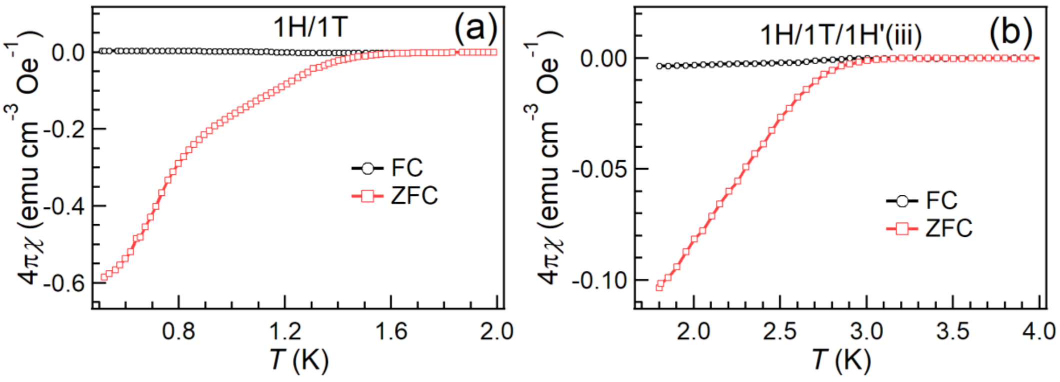

Figure S16. Superconducting volume fraction. The in-plane magnetic susceptibility ($4\pi\chi$) as a function of temperature for (a) 1H/1T-TaS_2 and (b) 1H/1T/1H'(iii)-TaS_2 crystals measured under an applied magnetic field of H=10 Oe. The superconducting volume fraction was estimated from the in-plane zero-field-cooled (ZFC) magnetic susceptibility to minimize the demagnetization effect inherent to the thin platelet geometry.

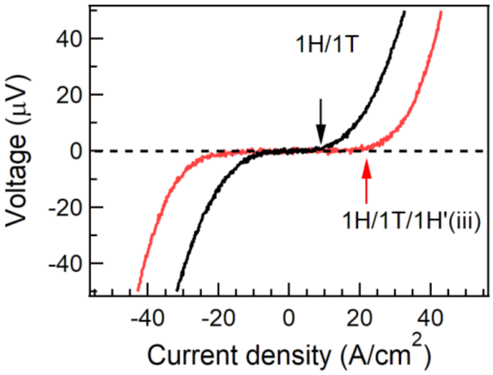

Figure S17. Current density-voltage (*J-V*) characteristics. *J-V* curves for 1H/1T-TaS_2 and 1H/1T/1H'(iii)-TaS_2 crystals collected at 1.6 K. The arrows guide the corresponding critical current densities.

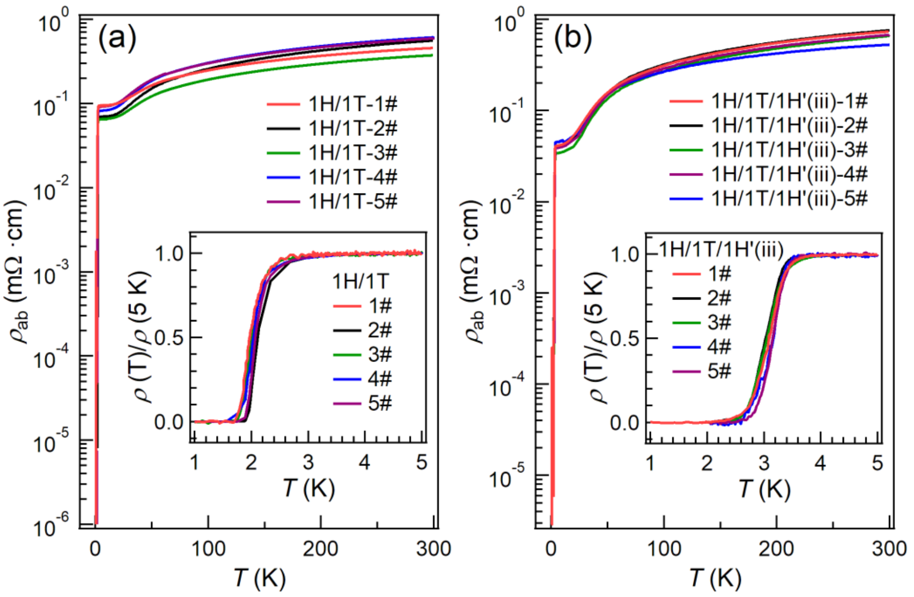

Figure S18. Reproducibility of superconducting transitions. Temperature-dependent in-plane resistivity for additional (a) five 1H/1T-TaS_2 and (b) five 1H/1T/1H'(iii)-TaS_2 crystals. The inset shows the close-up of resistivity close to superconducting transition. Measurements for five 1H/1T-TaS_2 crystals and one 1H/1T/1H'(iii)-TaS_2 crystal (1#) were performed using a separate PPMS system capable of cooling down to 0.5 K.

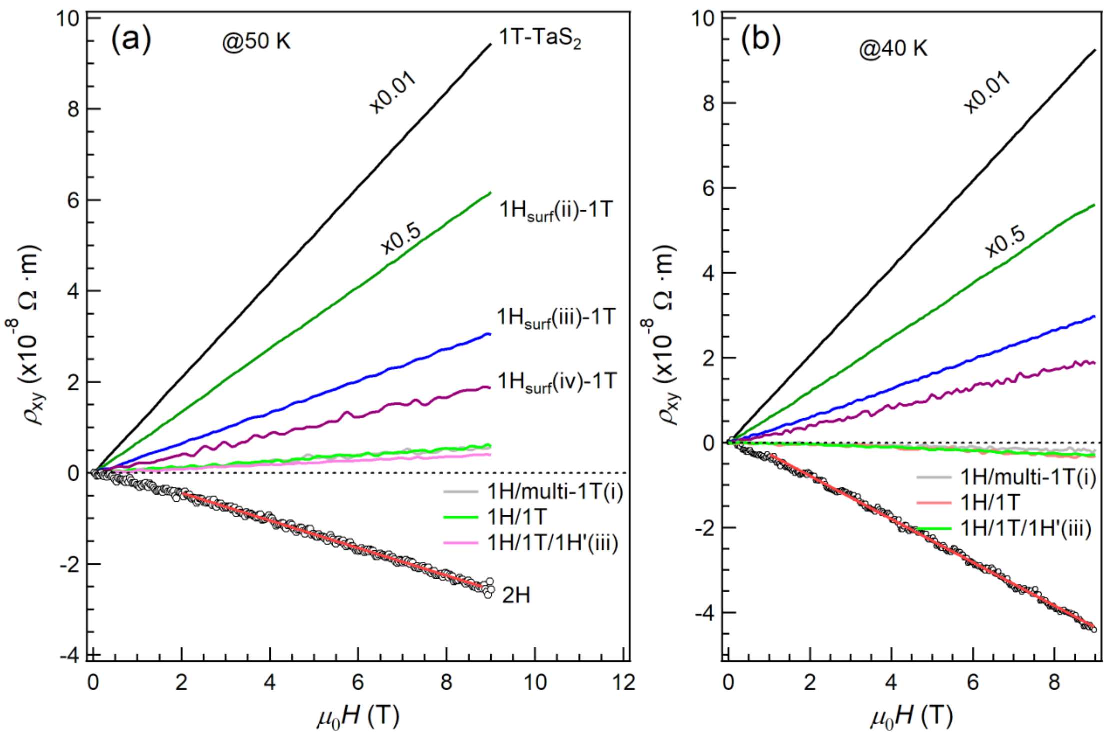

Figure S19. Hall resistivity of selected TaS_2 crystals. The Hall coefficient R_H extracted from linear fitting of Hall resistivity ρ_{xy} vs magnetic field at (a) 50 K and (b) 40 K. These data highlight the lower temperature (40-50 K) of R_H sign-change in 1H/multi-1T(i)-TaS_2, 1H/1T-TaS_2 and 1H/1T/1H'(iii)-TaS_2 crystals compared to naturally grown 2H-TaS_2 (~70 K).

Supplementary Note 7: Direct transformation from 1T-TaS_2 crystals to sandwiched 1H/1T/1H' superlattice, governed by two-tier sliding mechanism. Collective inter-layer sliding along $[\bar{1}\bar{2}0]$ (II) weakens inter-layer coupling and accumulates strain at the sliding interfaces. Pauli repulsion between the bottom S-atoms of the layer (iii) and top S-atoms of the underlying layer (iv) prevents further $[\bar{1}\bar{2}0]$ sliding. The strain is released through their oppositing intra-layer S-plane sliding, triggering individual-layer 1T-to-1H' and 1T-to-1H transition, respectively. The inter-phase electron transfer from intervening 1T layer to adjacent 1H and 1H' layers, together with electrostatic attraction between adjacent 1H' and 1H layers (iii and iv in X) suppresses misalignments and restores the vertical Ta-Ta alignment, ultimately forming 1H/1T/1H' superlattices (X).

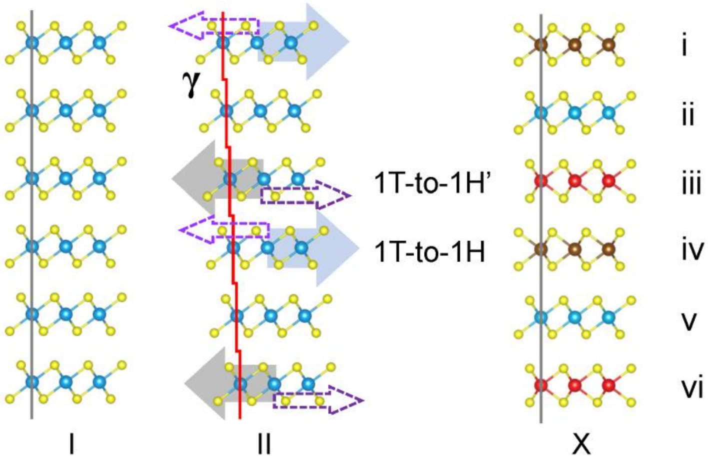

Figure S20. Structural transformation pathway. Schematic illustration of the structural transformation from 1T-TaS_2 (I) to inter-layer sliding arrangement (II), followed by formation of sandwiched 1H/1T/1H' superlattice (X) via individual-layer 1T-to-(1H or 1H') transitions, governed by two-tier sliding mechanism.

Supplementary Note 8. Potential sliding ferroelectricity in 1H/1T superlattice

The fundamental principle underlying sliding ferroelectrics requires that (i) the van der Waals system is non-centrosymmetric, and (ii) an equivalent polarization state can be achieved through a mirror operation and lateral inter-layer translation [12].

The 1H/1T superlattice is non-centrosymmetric and hosts an interfacial dipole. Two polarization states can, in principle, be obtained through mirror operations: mirror configuration I (horizontal plane reflection, Fig. S21b), and mirror configuration II (combined horizontal and vertical plane reflection, Fig. S21c).

As illustrated in Fig. S21d, mirror configuration I cannot be obtained through the proposed two-tier sliding mechanism. Achieving this configuration would require the formation of the 1T'-phase, the 60° rotational variant of the 1T-phase, which is not observed in our STEM measurements. However, mirror configuration II can be realized through intra-layer Ta-plane sliding associated with 1H-to-1H' transition (Fig. S21e).

Therefore, based on the experimentally observed structures, sliding ferroelectricity may be feasible in this system, but subjects further experimental investigation.

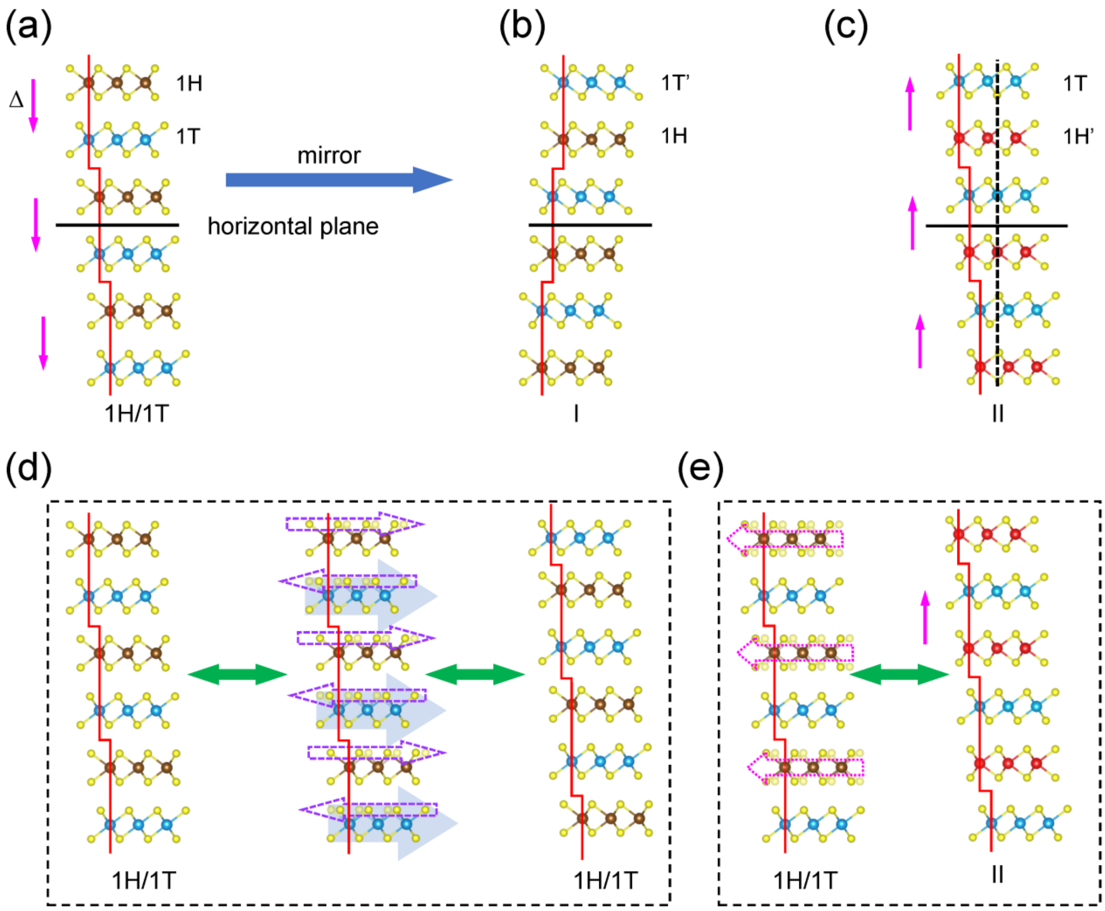

Figure S21. Schematic illustration of the structural pathways relevant to sliding ferroelectricity in the 1H/1T superlattice. (a) Stacking configuration of 1H/1T superlattice with an interfacial dipole. (b) Mirror configuration I obtained by reflection across the central horizontal plane. (c) Mirror configuration II involving horizontal and vertical plane reflection. Structural reconstruction based on the proposed two-tier sliding mechanism following (d) the stacking order reconstruction from configuration VII to VIII, and (e) intra-layer Ta-plane sliding, as illustrated in Fig. 2b and c of the main text.

References

1. Wang Y, Li Z and Luo X *et al.* Dualistic insulator states in 1T-TaS_2 crystals. *Nat Commun* 2024; **15**: 3425.
2. Liu Y, Ang R and Lu WJ *et al.* Superconductivity induced by Se-doping in layered charge-density-wave system 1T-$TaS_{2-x}Se_x$. *Appl Phys Lett* 2013; **102**: 192602.
3. Bu K, Zhang W and Fei Y *et al.* Possible strain induced Mott gap collapse in 1T-TaS_2. *Commun Phys* 2019; **2**: 146.
4. Shen S, Shao B and Wen C *et al.* Single-water-dipole-layer-driven reversible charge order transition in 1T-TaS_2. *Nano Lett* 2020; **20**: 8854–60.
5. Boix-Constant C, Mañas-Valero S and Córdoba R *et al.* Out-of-plane transport of 1T-TaS_2/graphene-based van der Waals heterostructures. *ACS Nano* 2021; **15**: 11898–907.
6. Duffey JR, Kirby RD and Coleman RV. Raman scattering from 1T-TaS_2. *Solid Stat Comm* 1976; **20**: 617–21.
7. Sugai S, Murase K and Uchida S *et al.* Studies of lattice dynamics in 2H-TaS_2 by Raman scattering. *Solid Stat Comm* 1981; **40**: 399-401.
8. Liu W, Duan Z and Zhang C *et al.* Experimental observations and density functional simulations on the structural transition behavior of a two-dimensional transition-metal dichalcogenide. *Sci Rep* 2020; **10**: 18255.
9. Chen K, Song M and Sun Y-Y *et al.* Defects controlled doping and electrical transport in TiS_2 single crystals. *Appl Phys Lett* 2020; **116**: 121901.
10. Di Salvo FJ, Bagley BG and Voorhoeve JM *et al.* Preparation and properties of a new polytype of tantalum disulfide ($4H_b$-TaS_2). *J Phys Chem Solids* 1973; **34**: 1357–62.
11. Meetsma A, Wiegers GA and Haange RJ *et al.* Structure of 2H-TaS_2. *Acta Cryst* 1990; **46**: 1598–99.
12. Yang Q, Wu M and Li J. Origin of two-dimensional vertical ferroelectricity in WTe_2 bilayer and multilayer. *J Phys Chem Lett* 2018; **9**: 7160–4.
